# Evolution of a plant growth-regulatory protein interaction specificity

**DOI:** 10.1038/s41477-023-01556-0

**Published:** 2023-10-30

**Authors:** Zhe Ji, Eric J. Belfield, Siyu Zhang, Jacques Bouvier, Shan Li, Jason Schnell, Xiangdong Fu, Nicholas P. Harberd

**Affiliations:** 1 Department of Biology, University of Oxford, Oxford, UK; 2 State Key Laboratory of Plant Cell and Chromosome Engineering, Institute of Genetics and Developmental Biology, Chinese Academy of Sciences, Beijing, P. R. China; 3 National Key Laboratory of Crop Genetics & Germplasm Enhancement and Utilization, Nanjing Agricultural University, Nanjing, PR China; 4 Department of Biochemistry, University of Oxford, Oxford, UK; 5 College of Life Sciences, University of Chinese Academy of Sciences, Beijing, P. R. China; 6 New Cornerstone Science Laboratory, Beijing, P. R. China

**Keywords:** Plant genetics, Plant evolution, Gibberellins, Plant signalling, Plant molecular biology

## Abstract

Specific protein–protein interactions (PPIs) enable biological regulation. However, the evolution of PPI specificity is little understood. Here we trace the evolution of the land-plant growth-regulatory DELLA–SLY1/GID2 PPI, revealing progressive increase in specificity of affinity of SLY1/GID2 for a particular DELLA form. While early-diverging SLY1s display relatively broad-range DELLA affinity, later-diverging SLY1s tend towards increasingly stringent affinity for a specific DELLA A’ form generated by the growth-promoting phytohormone gibberellin (GA). Our novel mutational strategy reveals amino acid substitutions contributing to the evolution of *Arabidopsis thaliana* SLY1 A’ specificity, also showing that routes permitting reversion to broader affinity became increasingly constrained over evolutionary time. We suggest that progressive affinity narrowing may be an important evolutionary driver of PPI specificity and that increase in SLY1/GID2-DELLA specificity enabled the enhanced flexibility of plant physiological environmental adaptation conferred by the GA-DELLA growth-regulatory mechanism.

## Abstract (web summary)

Ji and colleagues show that the specific angiosperm growth-governing DELLA–SLY1/GID2 protein interaction evolved from a broader ancestral affinity, suggesting affinity narrowing to be a general evolutionary driver of interaction specificity.

## Main

The DELLA-SLY1/GID2 (refs. ^1–6^) PPI regulates plant growth, survival of environmental adversity^7^ and resource assimilation that underpins terrestrial ecosystems and agriculture^8–10^. SLY1/GID2 is the F-box DELLA-specificity component of growth-promoting SCF^SLY1/GID2^ E3 ubiquitin ligase^3–6^. In angiosperms, gibberellin (GA) promotes DELLA–SCF^SLY1/GID2^ binding and resultant destruction of DELLA growth repressors, as follows. First, the GA-bound GID1 (refs. ^11–13^) GA receptor binds the DELLA N terminus. While the unbound N terminus is intrinsically unstructured, GA–GID1 binding induces folding^14^ and presumed conversion of the native (here called A) form of the C-terminal DELLA GRAS domain into an A’ alternative^2^. Although the molecular distinction between A and A’ is unclear, phosphorylation^4,5^ and/or reconfiguration of the GRAS domain structure may be causal. Nevertheless, the A to A’ transition is an important switch because SLY1/GID2 specifically binds the A’ GRAS domain, thus promoting DELLA destruction and growth.

**Extended Data Fig. 1:**
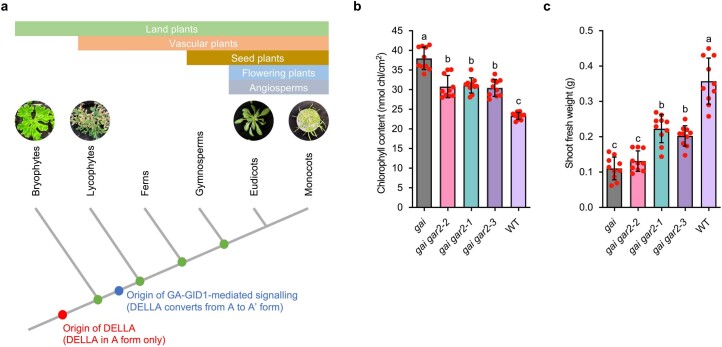
Land-plant phylogeny summary and suppression of *gai* phenotype by mutant *gar2* alleles. **a**, Summary of land-plant phylogeny showing major clades, origin of DELLA (red) and origin of GA-GID1-mediated signalling (blue). **b**, Mean chlorophyll contents, genotypes as shown, red dots indicate individual contents (n = 10), error bars indicate standard deviation, different letters (a-c) indicate significant difference (one-way ANOVA with Tukey’s test). **c**, Mean shoot fresh weights, genotypes as shown, red dots indicate individual weights (n = 10), error bars indicate standard deviation, different letters (a-c) indicate significant difference (one-way ANOVA with Tukey’s test). Source data

Angiosperms and GA-promoted growth both arose relatively recently, although at different times^2,15–18^ (Extended Data Fig. 1a). DELLAs probably evolved in the land-plant common ancestor^2,19^ from a GRAS protein of bacterial origin^20^, while functional GID1 GA receptors are exclusive to vascular plants (although some bryophytes have GID1-related proteins)^16–19^. Intriguingly, *Arabidopsis thaliana AtSLY1* (encoding AtSLY1) orthologues (for example, *Marchantia polymorpha MpSLY1* (ref. ^19^)) exist in liverworts, but not in mosses or hornworts (perhaps through gene loss during bryophyte evolution^21^). Nevertheless, the lack of functional bryophyte GID1s suggests that both GA–GID1-mediated DELLA destruction and SCF^SLY1/GID2^ A’ specificity first arose after bryophyte divergence (Extended Data Fig. 1a). Accordingly, bryophytes lack the active GA species that GID1 binds^2,22^.

To understand the origin of SLY1/GID2 A’ specificity, we first analysed variants with enhanced A affinity (reduced A’ specificity). Second, our analyses of SLY1s and DELLAs^23^ from early-diverging land plants revealed strong SLY1–DELLA A interactions. Together, our findings suggest that while ancestral SLY1 had dual A + A’ affinity, this affinity was progressively narrowed towards A’ specificity during land-plant evolution.

## Results

### Amino acid substitutions enhance AtSLY1 A affinity

**Fig. 1:**
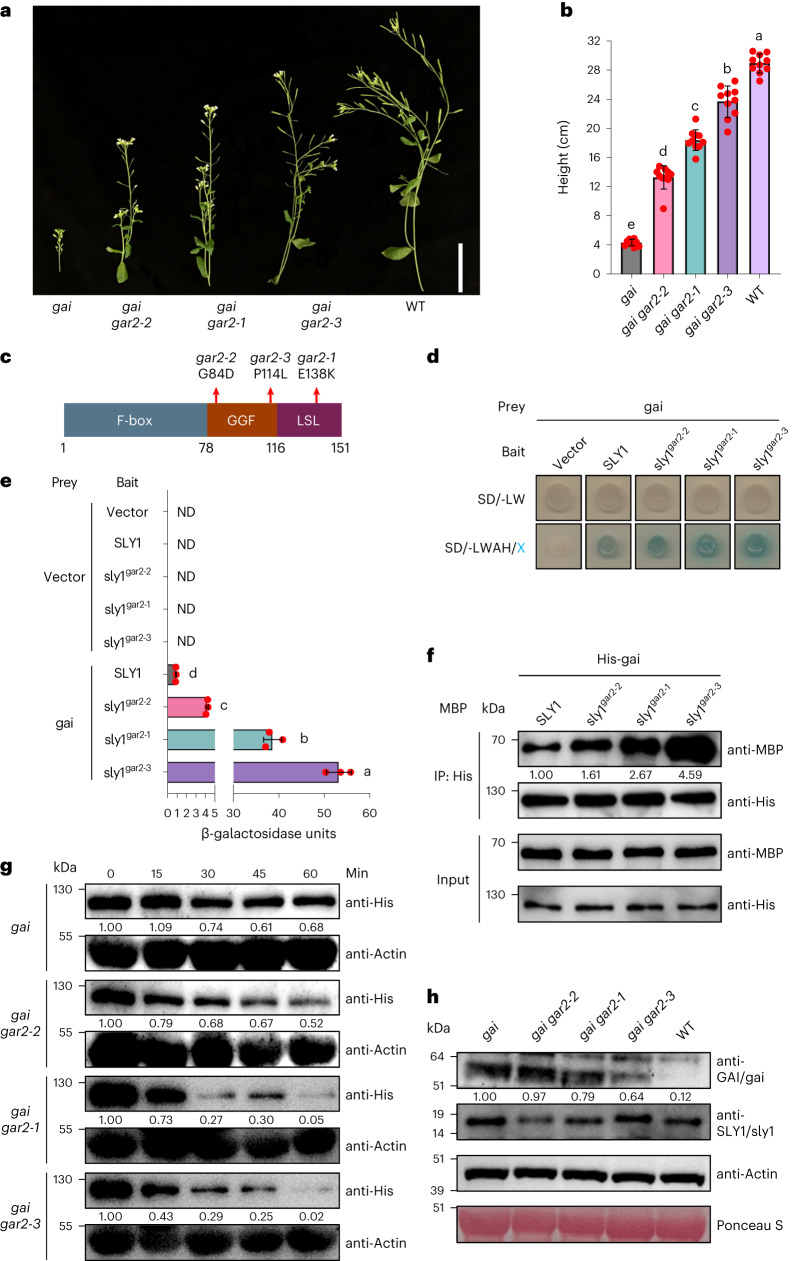
Mutant *gar2* alleles suppress the *gai* phenotype. **a**, *gar2* alleles variably suppress *gai*-conferred dwarfism. *gai* (*gai GAR2)* on far left, WT (*GAI GAR2*) on far right. Bolt stems cut from vegetative rosette. Scale bar, 5 cm. **b**, Mean (±s.d.) plant heights, genotypes as in **a**; red dots indicate individual heights (*n* = 10), different letters (a–e) indicate significant differences (one-way ANOVA with Tukey’s test). **c**, Amino acid substitutions encoded by *gar-2* alleles. Conserved F-box, GGF and LSL domains (positions 1–151) are indicated. **d**, Yeast 2-hybrid analysis of gai–Atsly1 interactions. Top line (SD/-LW) confirms double transformation (bait and prey constructs), bottom line (SD/-LWAH/X) indicates interaction: no detectable interaction in the absence of bait (empty vector), baseline gai-SLY1 interaction (medium blue), stronger gai–Atsly1^gar2^ interactions. **e**, Mean (±s.d.) yeast 2-hybrid interaction strengths, mutants as in **d**; note gradient of increasing interaction strength correlating with increase in plant height (**b**). ND, not detected; red dots indicate individual values (*n* = 3), different letters (a–d) indicate significant differences (one-way ANOVA with Tukey’s test). **f**, In vitro analysis of interactions between *E. coli*-expressed His-tagged gai and MBP-tagged Atsly1 variants. Anti-His serves as control and confirms that similar amounts of gai protein were used to ‘pull down’ a SLY1 or sly1 variant protein in each immunoprecipitation (IP) reaction, while anti-MBP shows how much SLY1 (quantified against anti-His, arbitrarily set at 1.00) or sly1 variant was pulled down. The increasing amounts of MBP-SLY1, MBP-sly1^gar2-2^, MBP-sly1^gar2-1^ and MBP-sly1^gar2-3^ detected suggests that the height (**b**) and yeast-based interaction (**e**) gradients reflect a gradient of increasing affinity. **g**, Destruction rates of *E. coli*-expressed His-gai in plant extracts quantified against Actin control (arbitrarily set at 1.00 for timepoint 0), genotypes as shown. While His-gai is appreciably degraded in the *gai* (*SLY1*) control by 60 min, *gar2* variant alleles confer faster degradation, with *gar2-3* being the fastest, consistent with the affinity gradient (**f**). **h**, Abundance of immuno-detectable gai (quantified against Actin, arbitrarily set at 1.00) or GAI and sly1 (or SLY1) in plant extracts (genotypes as shown). Anti-Actin and Ponceau S staining serve as loading controls. Source data

The *A. thaliana* mutant gai protein lacks the GAI DELLA domain^1^, does not bind GA–GID1 (ref. ^2^) and is therefore in the A form. Because AtSLY1 has low A affinity^5,6^, gai causes GA-insensitive dwarfism^1,24^ (Fig. 1a), a property enabling discovery of enhanced A affinity Atsly1 variants. For example, the E138 to K (E138K) amino acid substitution in Atsly1^gar2-1^ (encoded by *Atsly1*^*gar2-1*^) enhances A affinity, promotes gai destruction and suppresses *gai*-conferred dwarfism (Fig. 1a)^5,6^. Further *Atsly1* alleles (Fig. 1a) suppressed *gai* less (*Atsly1*^*gar2-2*^) or more (*Atsly1*^*gar2-3*^) than *Atsly1*^*gar2-1*^ (Fig. 1a,b and Extended Data Fig. 1b,c) due to G84D (Atsly1^gar2-2^) and P114L (Atsly1^gar2-3^) substitutions (Fig. 1c). Yeast 2-hybrid experiments next revealed the height gradient (Fig. 1a,b) to correspond with a gai affinity gradient (Atsly1^gar2-3^ > Atsly1^gar2-1^ > Atsly1^gar2-2^; Fig. 1d,e; AtSLY1 exhibits baseline gai affinity) also detected in vitro, with His-tagged gai pulling down increasing MBP-tagged Atsly1 amounts (Atsly1^gar2-3^ > Atsly1^gar2-1^ > Atsly1^gar2-2^; Fig. 1f) and partially reflected in plant extract gai destruction rates (Fig. 1g; although the Atsly1^gar2-1^/Atsly1^gar2-3^ differential was less clear than in Fig. 1e,f). In planta immuno-detected gai abundances were correspondingly reduced (Fig. 1h). Thus, E138K, G84D and P114L differentially enhance AtSLY1 affinity for gai DELLA A, in turn causing the height gradient (Fig. 1a,b). Furthermore, E138K, G84D and P114L exemplify distinct routes to enhancing AtSLY1 gai (A) affinity (reducing A’ specificity).

### Yeast-based discovery of Atsly1 variants

**Fig. 2:**
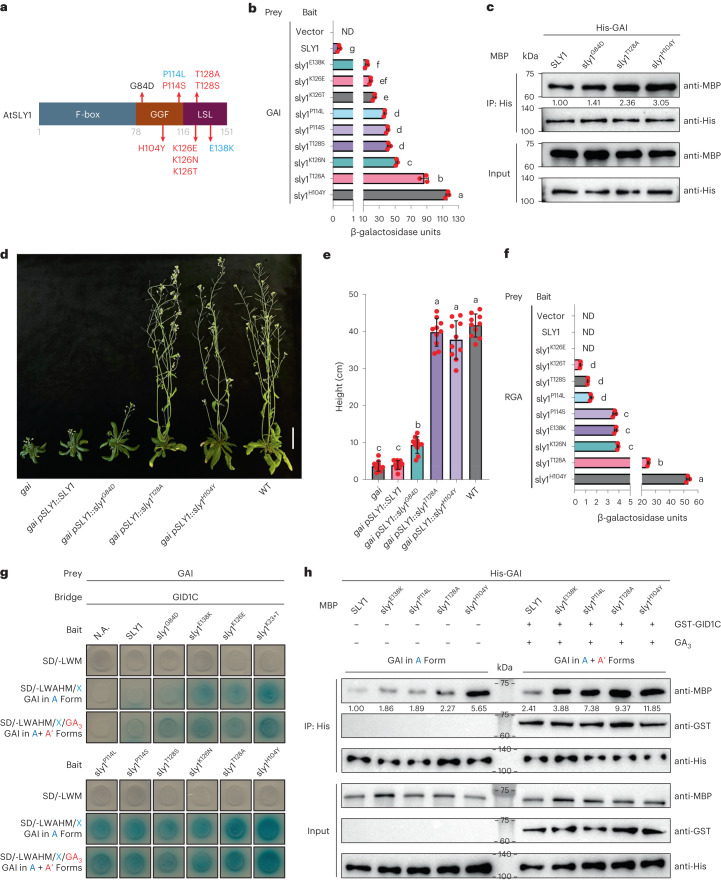
Yeast-based discovery of novel Atsly1 mutant proteins. **a**, Atsly1 substitutions shown in red were detected in yeast-based screens only, those in blue in both yeast-based and in planta screens. G84D (for reference) was detected in in planta screens only (see also Fig. 1c). **b**, Quantified (mean ± s.d.) yeast 2-hybrid AtSLY1–GAI interactions, Atsly1 variants as in **a**, arranged in a gradient of increasing interactions. Red dots indicate individual values (*n* = 3), different letters (a–g) indicate significant differences (one-way ANOVA with Tukey’s test). **c**, In vitro interactions between *E. coli*-expressed His-tagged GAI and MBP-tagged Atsly1 variants. Anti-His confirms similar amounts of GAI in each IP reaction, while anti-MBP shows how much SLY1 (quantified against anti-His, arbitrarily set at 1.00) or sly1 variant was pulled down. **d**, Transgenic expression of Atsly1 variants suppresses *gai* phenotype. Genotypes as shown, with *gai* (far left) and WT (far right) (for equivalence of expression, see Extended Data Fig. 2g). Scale bar, 5 cm. **e**, Mean (±s.d.) plant height, genotypes as in **d**, with non-transgenic WT and *gai*. Red dots indicate individual heights (*n* = 10), different letters (a–c) indicate significant differences (one-way ANOVA with Tukey’s test). **f**, Mean (±s.d.) yeast 2-hybrid AtSLY1–RGA interaction strengths, Atsly1 variants as in **a**. Red dots indicate individual values (*n* = 3), different letters (a–d) indicate significant differences (one-way ANOVA with Tukey’s test). **g**, Yeast 3-hybrid analysis of affinities for GAI A and A’ forms. GA promotes GAI–GID1C binding, converting GAI from A to A’. **h**, In vitro analysis of interactions between *E. coli-*expressed His-tagged GAI and MBP-tagged Atsly1 variants in the presence and absence of GST-GID1C and GA_3_. Anti-His confirms that similar amounts of GAI protein were used in each IP reaction, while anti-MBP and anti-GST show how much SLY1 (quantified against anti-His, arbitrarily set at 1.00) or sly1 variant and GID1C (if present) were pulled down. GA promotes GAI–GID1C binding, converting GAI from A to A’. Source data

**Extended Data Fig. 2:**
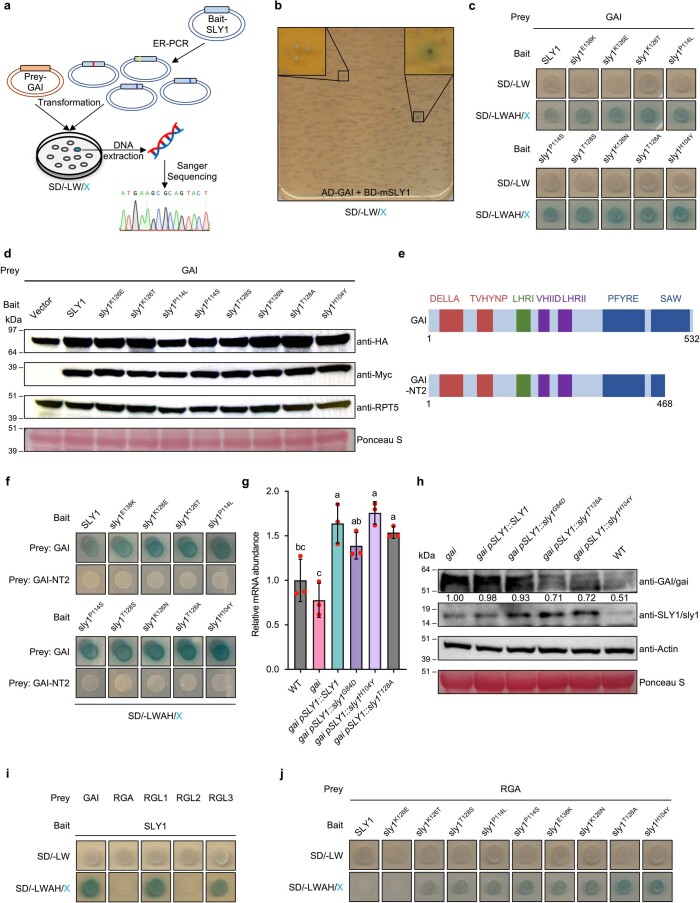
Yeast-based discovery of novel Atsly1 mutant proteins. **a**, Combined EP-PCR/yeast-2-hybrid screen for Atsly1 variants having increased AtGAI affinity. Different Atsly1 amino acid substitution variants (represented by different colours) are generated via error prone PCR, then used as bait against an AtGAI prey in a yeast-2-hybrid screen. Colonies exhibiting increased interaction are blue (rather than white; see also b). DNA sequencing then reveals interaction-increasing amino acid substitutions (see Methods). **b**, Example yeast-2-hybrid screen plate showing a blue colony (also in close-up panel) amongst whites (also in close-up). **c**, Yeast-2-hybrid analysis of interactions between GAI and Atsly1 variants. Top line (SD/-LW) confirms double transformation with bait and prey constructs, bottom line (SD/-LWAH/X) indicates interaction: baseline interaction between GAI and SLY1 (medium blue), stronger interactions between GAI and Atsly1 variants. **d**, Accumulation of immuno-detectable AtSLY1 and Atsly1 variants in yeast. GAI is tagged with HA, SLY1 and sly1 variants are tagged with Myc. RPT5 and Ponceau S provide loading controls. **e**, GAI compared with C-terminally truncated GAI-NT2. **f**, SLY1 and sly1 variant interactions with GAI and GAI-NT2 (as shown). AtSLY1^6^ and all Atsly1 variants interact with GAI but not with GAI-NT2. The increased interaction of Atsly1 variants is thus not due to increased general ‘stickiness’. **g**, Mean relative AtSLY1 mRNA abundances in selected transgenic lines (genotypes as shown), with non-transgenic WT and *gai*. Red dots indicate individual values (n = 3), error bars indicate standard deviation, different letters (a-c) indicate significant difference (one-way ANOVA with Tukey’s test). **h**, Abundance of immuno-detectable gai (quantified against Actin, arbitrarily set at 1.00) or GAI and sly1 (or SLY1) in plant extracts (genotypes as shown). Anti-Actin and Ponceau S staining serve as loading controls. **i**, Interactions between AtSLY1 and *Arabidopsis* DELLAs GAI, RGA, RGL1, RGL2 and RGL3. **j**, Interactions between AtSLY1 or Atsly1 variants and RGA. Source data

Next, error-prone PCR (EP-PCR)-generated Atsly1 variants were screened in yeast for enhanced GAI affinity (Methods and Extended Data Fig. 2a,b). GAI is a yeast proxy for in planta A because, in the absence of GA or GID1, A’ cannot arise. The screen revealed 9 substitutions (Fig. 2a and Supplementary Table 1), 2 of which (E138K and P114L) replicate Atsly1^gar2-1^ and Atsly1^gar2-3^ substitutions (Figs. 1a–c and 2a), thus validating yeast AtSLY1–GAI interactions as in planta proxy. The remaining 7 yeast-selected Atsly1 variants are novel, and their yeast-reported enhanced A affinities (Fig. 2b and Extended Data Fig. 2c) are unlikely due to increased accumulation (Extended Data Fig. 2d) or non-specific binding (Extended Data Fig. 2e,f).

Yeast-reported and in vitro Atsly1 variant A affinity enhancement ranges from weak (E138K; Atsly1^gar2-1^) to strong (Atsly1^H104Y^; Fig. 2b,c and Extended Data Fig. 2c). Constructs expressing Atsly1 variants (Extended Data Fig. 2g) conferred corresponding graduated suppression of *gai*-conferred dwarfism: Atsly1^G84D^ caused mild height increase, while Atsly1^T128A^ and Atsly1^H104Y^ conferred tall (similar to wild type (WT)) phenotypes (Fig. 2d,e). This height gradient was reflected in gai abundance reductions (Extended Data Fig. 2h), confirming that the yeast-selected Atsly1 variants promoted gai degradation and demonstrating their biological (in planta) relevance.

Importantly, Atsly1 variant A affinity enhancement is not restricted to GAI (or gai). Of the 5 *A. thaliana* DELLAs^2^, AtSLY1 displays baseline GAI, RGL1 and RGL3, but not RGA or RGL2 affinity (Extended Data Fig. 2i). Interestingly, an Atsly1 RGA A affinity gradient (Fig. 2f and Extended Data Fig. 2j) essentially replicates (despite quantitative reduction) the GAI gradient (Fig. 2b), suggesting general DELLA A affinity enhancement. Furthermore, increased A affinity is not detectably associated with reduced A’ affinity. DELLAs retain GA responsivity^5,6^ in *sly1*^*gar2-1*^, indicating that E138K enhances A affinity without reducing affinity for the GA-promoted A’ form. We showed similar retention of A’ affinity in additional Atsly1 variants. Employing a yeast 3-hybrid approach with GAI (prey), SLY1/sly1 (bait) and GID1C (bridge) partners, we expected GA to cause GAI binding to GA-GID1C, thus generating A’ and (because SLY1 A’ affinity is strong) detectably increasing SLY1-GAI interactions (as seen in Fig. 2g). In contrast, while reduced A’ affinity (if any) would be expected to reduce GAI–Atsly1 interactions in response to GA, this was detected neither in yeast (Fig. 2g) nor in complementary ‘pull-down’ experiments (Fig. 2h). Thus, the enhanced A affinity of Atsly1 variants is not detectably associated with reduced A’ affinity. Rather, the high-specificity A’ affinity of AtSLY1 is transformed in Atsly1 variants into broader A + A’ affinity.

### Atsly1 substitutions alter the DELLA-interacting region

**Fig. 3:**
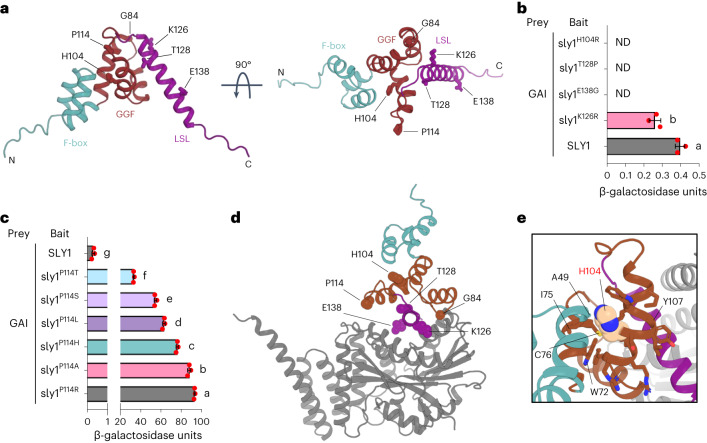
Structural and selective consideration of Atsly1 mutant proteins. **a**, AlphaFold AtSLY1 structure prediction shown in 90^o^ rotation and with helical F-box (cyan), core helical GGF (brown) and outward-facing helical LSL domains (purple) (see also Extended Data Fig. 3a–c). Amino-acid side chains of residues targeted by substitutions in Atsly1 mutant proteins (for example, H104) are shown as sticks and labelled. The position of G84 is indicated by a sphere. The N-terminal 25 residues, which are predicted to be unstructured, are not shown. **b**, Mean (±s.d.) strengths of yeast 2-hybrid interactions between AtSLY1 (or indicated variants) and GAI. Red dots indicate individual values (*n* = 3), different letters (a, b) indicate significant difference (two-sided Student’s *t*-test). *P* = 0.0044. **c**, Mean (±s.d.) strengths of yeast 2-hybrid interactions between AtSLY1 (or indicated variants) and GAI. Red dots indicate individual values (*n* = 3), different letters (a–g) indicate significant differences (one-way ANOVA with Tukey’s test). **d**, AlphaFold prediction of the AtSLY1–GAI complex structure. Amino-acid residues targeted by substitutions in Atsly1 mutant proteins are shown as spheres and labelled (see also Extended Data Fig. 4e,f). N-terminal residues of SLY1 and GAI predicted to be unstructured are not shown. **e**, AlphaFold AtSLY1–GAI structural prediction showing H104 intramolecular contacts within AtSLY1. The H104 side chain is in van der Waals spheres and side chains for residues with atoms ≤5 Å from any H104 atom are shown as sticks. GAI is shown as a grey ribbon. All residue contacts ≤5 Å are within SLY1 and, apart from the F-box residue A49, all are within the GGF region and include W72, I75 and C76. A ‘bulge’ visible at Y107 in the helix is due to formation of a π-helix turn starting at H104. Source data

**Extended Data Fig. 3:**
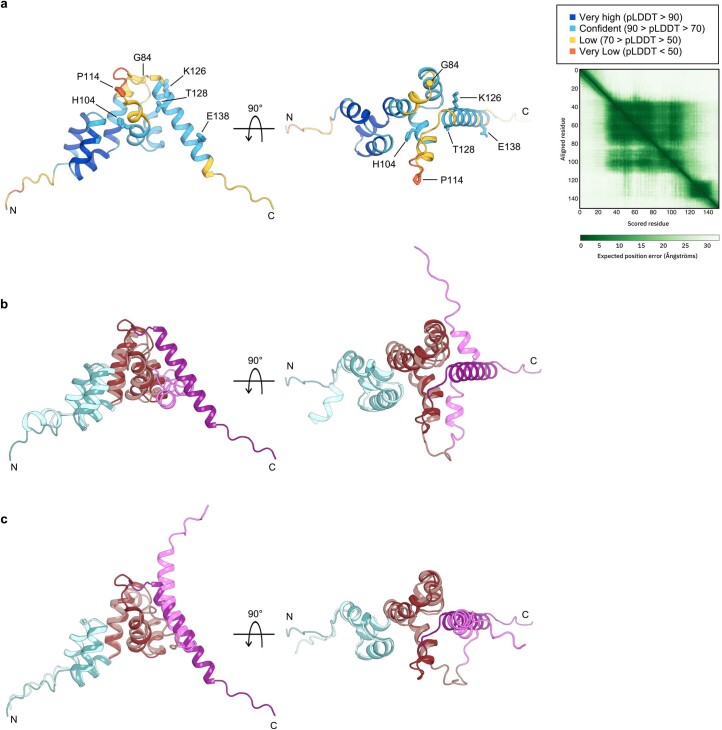
AtSLY1 structural predictions. **a**, AlphaFold AtSLY1 structural prediction shown in 90^o^ rotation and color-coded according to pLDDT confidence levels. At right is the predicted aligned error for the structural model. The colour at position (x, y) indicates AlphaFold’s expected position error at residue x, when the predicted and true structures are aligned on residue y. **b**, AtSLY1 structural alignment between the AlphaFold model (darker shades) and the top RoseTTAFold (lighter shades) model shown in 90˚ rotation and color-coded according to region: helical F-box (cyan), GGF (brown), and LSL helix (purple). The N-terminal 25 residues, which are predicted to be unstructured, are not shown. **c**, AtSLY1 structural alignment between the AlphaFold (darker shades) and ESMfold (lighter shades) models shown in 90˚ rotation and color-coded as in b. The N-terminal 25 residues, which are predicted to be unstructured, are not shown. Source data

AtSLY1 has N-terminal F-box (SCF-tethering) and C-terminal DELLA-interacting GGF and LSL domains^5,6^. AlphaFold^25,26^ predicts a core GGF region of three α-helices, a ~9-residue linker and C-terminal LSL helical regions (Fig. 3a and Extended Data Fig. 3a), with RoseTTAfold^27^ and ESMfold^28^ predictions (Extended Data Fig. 3b,c) broadly agreeing. Interestingly, predicted LSL helix and GGF domain alignment errors are large, and predicted LSL helix structures and positions relative to the GGF domain differ (Extended Data Fig. 3a–c). Nevertheless, all models predict an outward-facing LSL helix, consistent with a likely role in direct AtSLY1–DELLA interactions.

**Extended Data Fig. 4:**
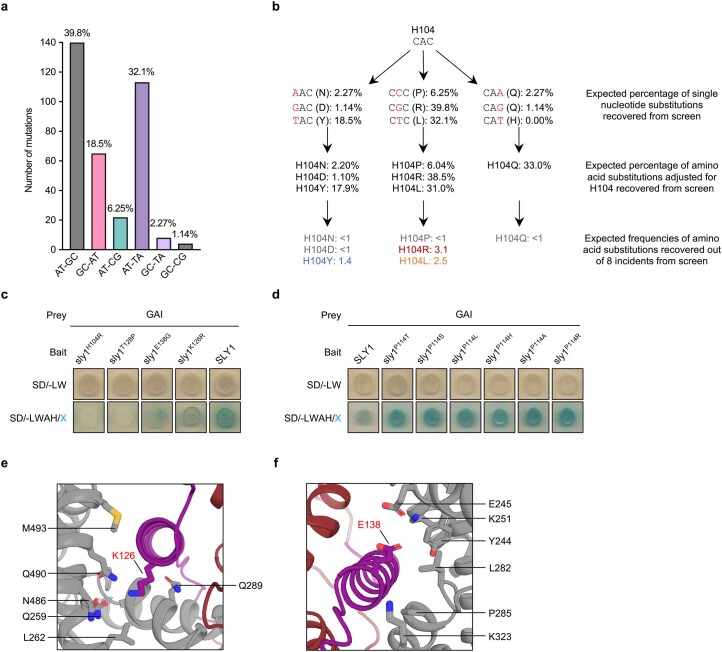
Expected frequencies and GAI interactions of Atsly1 variants. **a**, Frequencies of specific nucleotide substitutions generated by EP-PCR. **b**, Example calculation of expected frequencies of EP-PCR generated amino acid substitutions at position H104 of AtSLY1 in the absence of selection. Individual nucleotide substitutions are shown in red. Resultant expected amino acid substitutions (percentages) and actual numbers (out of 8 events) are as shown. Whilst H104R (red) and H104L (orange) are expected most frequently, H104Y (blue) predominates. **c, d**, Yeast-2-hybrid analysis of interactions between GAI and Atsly1 variants (as shown). **e**, AtSLY1 K126 intermolecular contacts in the AlphaFold predicted complex with GAI. The K126 sidechain of AtSLY1 (purple) and the sidechains of all GAI (grey) residues with atoms ≤5 Å from K126 are shown as sticks. **f**, As in **e**, but for E138.

All Atsly1 substitutions alter GGF/LSL residues (Figs. 1c,2a and 3a). Our probabilistic considerations next determined whether they alter AtSLY1–DELLA interactions via function-critical residue loss, or via function-altering replacement. For example, we recovered 8 independent H104Y substitutions (Supplementary Table 1). However, single-nucleotide mutation of the H104 codon can theoretically cause 1 of 7 substitutions (H to D, L, N, P, Q, R or Y). Using EP-PCR-generated mutation frequencies (Extended Data Fig. 4a), we determined expected amino acid substitution frequencies, finding that without selection, H104R should predominate (Extended Data Fig. 4b) and that recovery of 8 H104Y substitutions suggests selection (*χ*^2^ = 36.8, *P* < 0.01; Supplementary Table 2). Presumably, H104Y enhances AtSLY1–GAI interactions, while other substitutions do not (see also below). K126 (*χ*^2^ = 39.7, *P* < 0.01; Supplementary Table 2) and E138 (*χ*^2^ = 26.9, *P* < 0.01; Supplementary Table 2) substitutions also indicate selection, with possible weak preference at T128 (*χ*^2^ = 9.44, *P* < 0.05; Supplementary Table 2) and no preference detectable at P114 (Supplementary Table 2). We suggest that P114 substitutions cause function-critical residue loss, while function-altering replacements at H104, K126, E138 and possibly T128 increase AtSLY1–DELLA interactions.

Testing these suggestions, we showed that expected (unselected) predominant substitutions at H104, K126, T128 or E138 did not enhance baseline AtSLY1–GAI interactions and indeed reduced (Atsly1^K126R^) or abolished (Atsly1^H104R^, Atsly1^T128P^ and Atsly1^E138G^; Fig. 3b and Extended Data Fig. 4c) them. This confirmed the specificity of the selected H104, K126, T128 and E138 substitutions and the importance of these sites to AtSLY1–GAI interactions. In contrast, all possible P114 substitutions enhanced AtSLY1–GAI interactions (Fig. 3c and Extended Data Fig. 4d), again indicating that loss of function-critical P114 confers phenotypic change.

Both AtSLY1 intramolecular and AtSLY1–GAI intermolecular interactions probably influence AtSLY1–GAI affinity. An AlphaFold-multimer AtSLY1–GAI complex model (Fig. 3d) predicts that although the GRAS domain directly interacts with the LSL helix, some residues targeted in Atsly1 variants are buried within AtSLY1, distant from the GAI interface. For example, H104, buried within the GGF domain, has predicted intramolecular AtSLY1 contacts but no predicted GAI intermolecular contacts (Fig. 3e). H104Y therefore probably enhances AtSLY1–GAI interactions via internal effects, perhaps changing the relative orientation or stability of the GGF/LSL interface. Accordingly, the variation in predicted LSL structure (Extended Data Fig. 3a–c; see above) may reflect functionally relevant conformational dynamics. The model (Fig. 3d) further indicates that the LSL helix K126 (Extended Data Fig. 4e) and E138 (Extended Data Fig. 4f) substitutions directly affect interatomic electrostatic contacts at the AtSLY1–GAI interface, whereas T128 points away from it and towards the GGF/LSL domain interface. Of the two remaining substituted positions, G84 is on the surface of the GGF region and close to both the intermolecular GAI interface and the intramolecular LSL helix interface, suggesting both direct and indirect effects, while P114 is in an unstructured loop connecting the GGF and LSL helices in predicted structures of AtSLY1 alone, but terminates the third GGF helix in the predicted AtSLY1–GAI complex. Prolines are uniquely potent in terminating helices, perhaps explaining why all observed P114 substitutions enhance GAI affinity.

### Basal SLY1s exhibit broad-range DELLA affinity

**Fig. 4:**
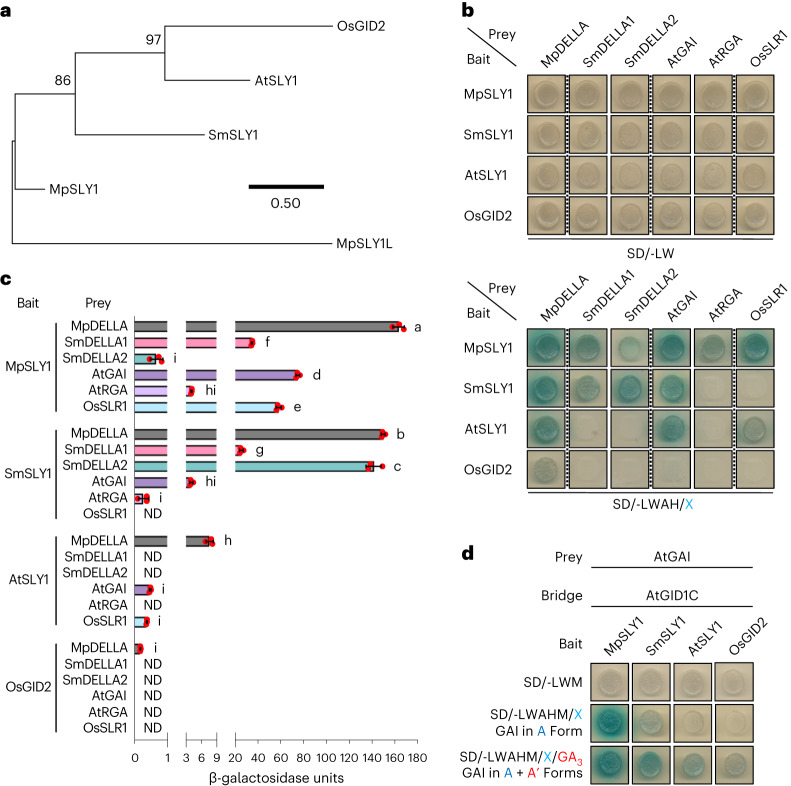
Basal SLY1s exhibit broader DELLA affinities. **a**, Phylogenetic tree showing SLY1/GID2 orthologues from *M. polymorpha* (MpSLY1), *S. moellendorfii* (SmSLY1), *A. thaliana* (AtSLY1) and rice (*O. sativa*; OsGID2) with MpSLY1L (MpSLY1-like) outgroup. The tree was constructed in MEGA11 using the maximum-likelihood method and the JTT matrix-based model. The percentage of trees in which the associated taxa clustered together (bootstrap value) is shown next to the branches (out of 500 bootstrap replicates). The tree is drawn to scale, with branch lengths measured as the number of substitutions per site. For a more comprehensive phylogeny, see Extended Data Fig. 5. **b**, Interactions between SLY1 and DELLA orthologues. **c**, Mean (±s.d.) yeast 2-hybrid SLY1 orthologue–GAI interaction strengths; note gradient of increasing interaction strength ranging from OsGID2 to MpSLY1. Red dots indicate individual values (*n* = 3), different letters (a–i) indicate significant differences (one-way ANOVA with Tukey’s test). **d**, Yeast 3-hybrid determination of relative affinities of SLY1 orthologues for GAI A and A’ forms. Source data

**Extended Data Fig. 5:**
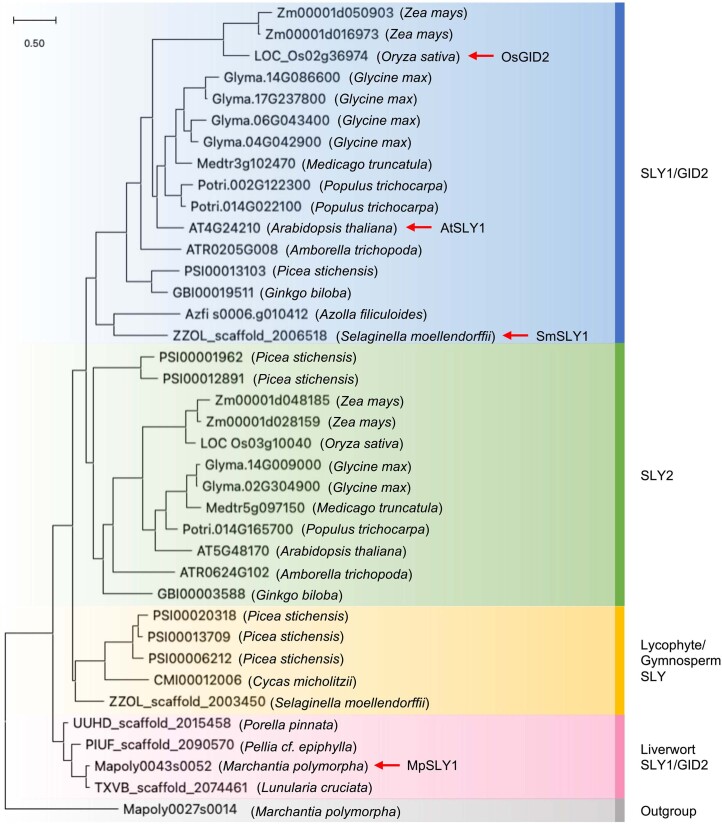
SLY1 orthologue phylogenetic tree. Comprehensive phylogenetic analysis of SLY1 orthologues from diverse species, showing major SLY1/GID2, SLY2, Lycophyte/Gymnosperm SLY and Liverwort SLY1/GID2 clades. MpSLY1, SmSLY1, AtSLY1 and OsGID2 are highlighted (red arrows). A related sequence (Mapoly0027s0014/MpSLY1L from *M. polymorpha*) is used as outgroup. The tree was constructed in MEGA11 using the Maximum Likelihood method and JTT matrix-based model. The tree is drawn to scale, with branch lengths measured in the number of substitutions per site.

**Extended Data Fig. 6:**
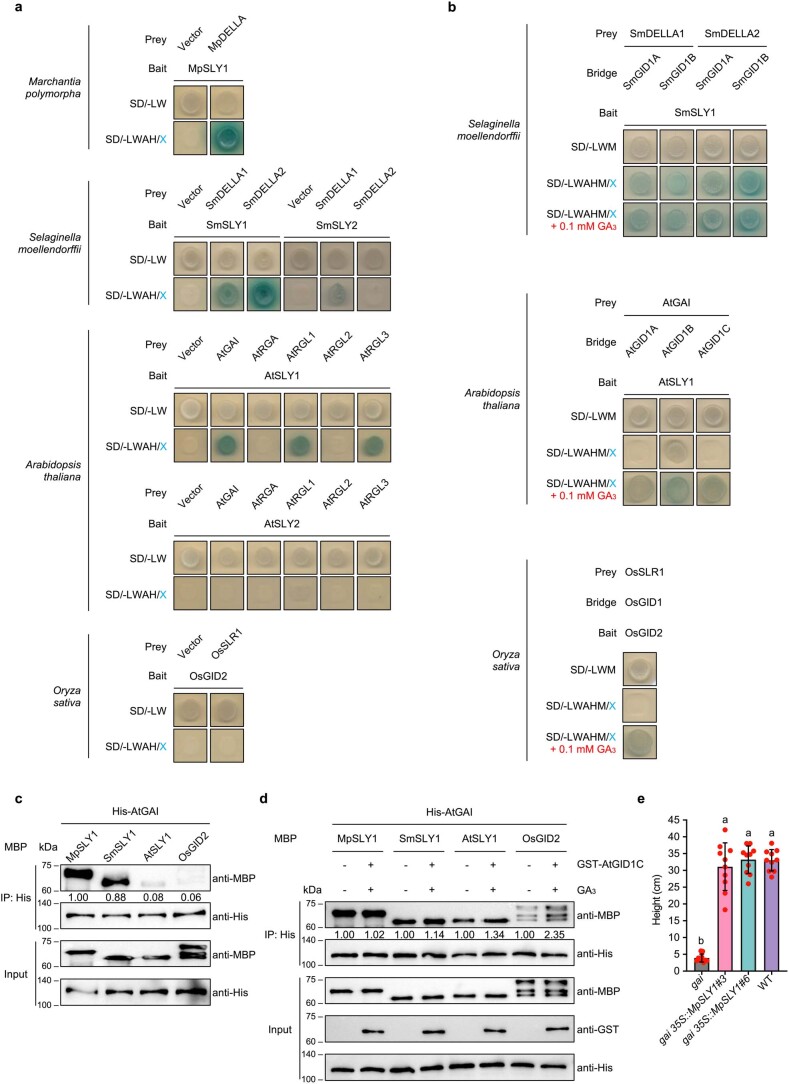
Basal SLY1s exhibit broader DELLA affinities. **a**, Yeast-2-hybrid analysis of interactions between SLY1 and DELLA orthologues from various species (as indicated). **b**, Yeast-3-hybrid analysis of interactions between SLY, GID1 and DELLA orthologues from various species (as indicated). **c**, *in vitro* analysis of interactions between *E.coli*-expressed His-tagged AtGAI and MBP-tagged SLY1/GID2s. Anti-His serves as control and confirms that similar amounts of AtGAI protein were used to ‘pull down’ a SLY1/GID2 protein in each immunoprecipitation (IP) reaction, whilst anti-MBP shows how much SLY1/GID2 (quantified against anti-His, with MpSLY1 arbitrarily set at 1.00) was pulled down. **d**, *in vitro* analysis of interactions between *E.coli*-expressed His-tagged AtGAI and MBP-tagged SLY1/GID2s in the presence and absence of GST-AtGID1C and GA_3_. Anti-His serves as control and confirms that similar amounts of AtGAI protein were used to ‘pull down’ a SLY1/GID2 protein in each immunoprecipitation (IP) reaction, whilst anti-MBP shows how much SLY1/GID2 (quantified against anti-His, with SLY1/GID2 in the absence of GST-GID1C and GA_3_ arbitrarily set at 1.00) was pulled down. **e**, Mean plant heights, red dots indicate individual heights (n = 10), error bars indicate standard deviation, different letters (a and b) indicate significant difference (one-way ANOVA with Tukey’s test). Source data

We next determined whether Atsly1 properties reflect SLY1 evolution. Previous analyses identified duplicate *A. thaliana AtSLY1* and *AtSLY2* genes, with encoded AtSLY1 dominating AtSLY2 in GA signalling^5,29^. Our SLY1 phylogeny reflected land-plant evolution, revealing a bryophyte (liverwort) SLY1 clade and two more-recent SLY1/GID2 (containing *AtSLY1*) and SLY2 (containing *AtSLY2*) clades, which separated before lycophyte divergence (Fig. 4a and Extended Data Fig. 5). Broadly, while the liverwort *Marchantia polymorpha* genome encodes MpSLY1, MpDELLA, but no functional GID1 (GA receptor orthologue), the lycophyte *Selaginella moellendorfii* genome encodes SmSLY1, SmSLY2, SmDELLA1, SmDELLA2 and SmGID1 representatives^2,16–19^ (Extended Data Fig. 1a). Comparing the yeast DELLA A form affinities of MpSLY1, SmSLY1, SmSLY2, AtSLY1, AtSLY2 and GID2 (rice (monocot angiosperm *Oryza sativa*) AtSLY1 orthologue), we first found that SLY2 clade representatives (SmSLY2, AtSLY2) lacked detectable DELLA interactions (Extended Data Fig. 6a) and excluded them from further analysis. Next, we detected progressively reduced SLY1 A affinity: MpSLY1-MpDELLA > SmSLY1-SmDELLA1/SmDELLA2 > AtSLY1–GAI/RGA > GID2-SLR1 (Fig. 4b,c; SLR1 is rice DELLA), suggesting progressive evolutionary reduction of strong ancestral SLY1 A affinity. Furthermore, while MpSLY1 interacts with all DELLAs tested, and SmSLY1 interacts with a reduced subset, AtSLY1 selectively interacts with MpDELLA, GAI (not RGA) and SLR1, while GID2 exhibits weak interaction with MpDELLA only (Fig. 4b,c). We conclude that basal SLY1s (MpSLY1; SmSLY1) have broad-ranging affinity for various DELLAs (perhaps reflecting ancestral SLY1) and that affinity became more stringent, more species and A’-specific during evolution. In addition, because GID2 interacts weakly with MpDELLA, DELLA-SLY1 co-evolution is a possibility, although this was not investigated further.

In further experiments, MpSLY1 exhibited no detectable differential A vs A’ affinity, SmSLY1 exhibited mild preference for A’, while A’ preference was yet stronger in angiosperm AtSLY1 and GID2 (Fig. 4d and Extended Data Fig. 6b–d). Because transgenic expression of MpSLY1 suppresses *Arabidopsis gai* phenotype (Extended Data Fig. 6e), these observations are biologically relevant, suggesting that ancestral SLY1 had strong A + A’ affinity (despite the ancestral absence of A’) and that while A affinity declined during evolution, A’ affinity was retained. Thus, while the relative A’ specificity of angiosperm SLY1s is probably due to evolutionary narrowing of broad-range A + A’ affinity, the partially restored A + A’ affinities of Atsly1 variants exemplify ‘evolutionary revertant’ phenotypes.

### Evolutionarily revertant Atsly1 variant substitutions

**Fig. 5:**
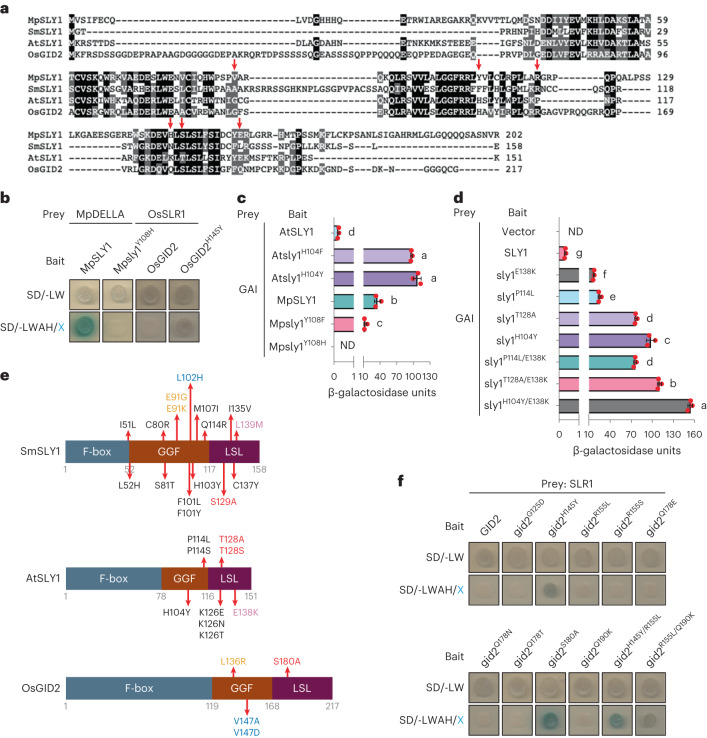
Evolutionary constraints on SLY1 reversion routes. **a**, Alignment of amino acid sequences of SLY1 orthologues. Black highlights identity, grey highlights residue similarity, red arrows indicate sites in AtSLY1 targeted by selected amino acid substitutions (and equivalent sites in MpSLY1, SmSLY1 and OsGID2). For more comprehensive alignment, see Extended Data Fig. 7. **b**, Interactions between MpSLY1 or OsGID2 (or indicated variants) and MpDELLA or OsSLR1. **c**, Mean (±s.d.) strengths of yeast 2-hybrid interactions between AtSLY1 (or indicated variants) or MpSLY1 (or indicated variants) and GAI. Red dots indicate individual values (*n* = 3), different letters (a–d) indicate significant differences (one-way ANOVA with Tukey’s test). **d**, Mean (±s.d.) strengths of yeast 2-hybrid interactions between AtSLY1 (or indicated variants) and GAI. Red dots indicate individual values (*n* = 3), different letters (a–g) indicate significant differences (one-way ANOVA with Tukey’s test). **e**, Comparison of sites where amino acid substitutions confer increased affinity for the DELLA A form. Data from EP-PCR/yeast 2-hybrid screens. For SmSLY1, mutants were initially selected for increased affinity for GAI, then validated with SmDELLA1. For AtSLY1, substitutions were as previously described (Fig. 2a). For OsGID2, mutants were initially selected for increased affinity for GAI, then validated with the rice DELLA SLR1. Targeted amino acids occupying equivalent positions are indicated with the same colour. Specifically, SmSLY1 E91 is equivalent to OsGID2 L136 (orange); SmSLY1 L102 is equivalent to OsGID2 V147 (blue); SmSLY1 L139 is equivalent to AtSLY1 E138 (purple); SmSLY1 S129 is equivalent to AtSLY1 T128 and OsGID2 S180 (red). All other targeted positions (black) are unique to SmSLY1 or AtSLY1. **f**, Interaction of indicated OsGID2 and Osgid2 variants with SLR1. Source data

**Extended Data Fig. 7:**
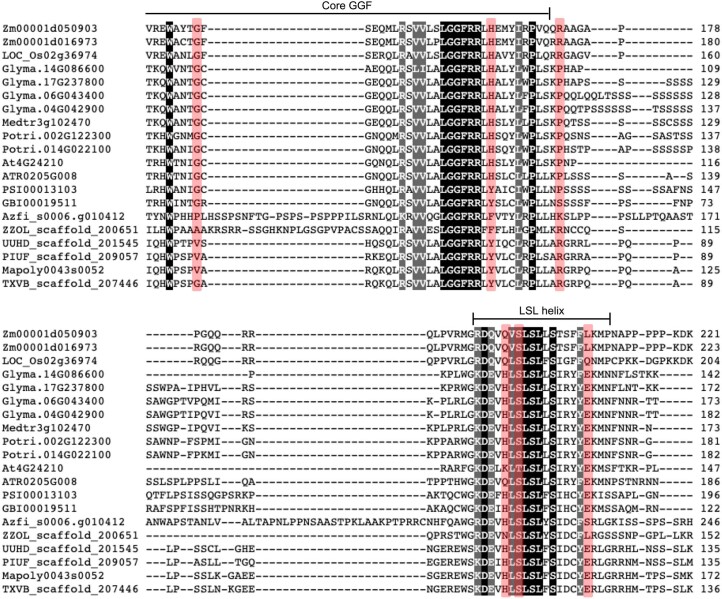
Sequence alignment of selected SLY1 orthologues. Alignment of SLY1 orthologue amino-acid sequences from various species (as indicated) showing regions of conservation (GGF and LSL domains) and positions affected in screens for enhanced A affinity. Black highlights identical amino-acids, grey highlights amino acids that are similar. Red boxes highlight sites in AtSLY1 targeted by selected amino-acid substitutions (and equivalent sites in SLY1s from SLY1/GID2 and liverwort SLY1/GID2 clades in Extended Data Fig. 5).

Although within or close to the conserved GGF/LSL domains (Fig. 5a and Extended Data Fig. 7), Atsly1 substitutions often target recently acquired residues, such as K126 (which is unique to AtSLY1; Extended Data Fig. 7). Similarly, the recent T128 is S in almost all (including basal) sequences (Extended Data Fig. 7) and is restored in Atsly1^T128S^. While P114R strongly enhances A affinity, R is at the equivalent position in basal SLY1s (Extended Data Fig. 7). Finally, H104 was probably substituted before angiosperm divergence (104 or equivalent position is H in all angiosperms, including the basal *Amborella*; Extended Data Fig. 7). Thus, the phenotype-changing substitutions at K126, T128, P114 and H104 are all evolutionarily revertant substitutions. Quantitatively, H104Y confers an A affinity (Fig. 2b) roughly equivalent to that of MpSLY1 (Fig. 4c), indicating a major role for H104Y during GA-DELLA signalling evolution. We suggest that AtSLY1 evolved by suppressing ancestral A affinity, with some Atsly1 substitutions partially restoring A affinity by reversing evolutionary substitutions.

**Extended Data Fig. 8:**
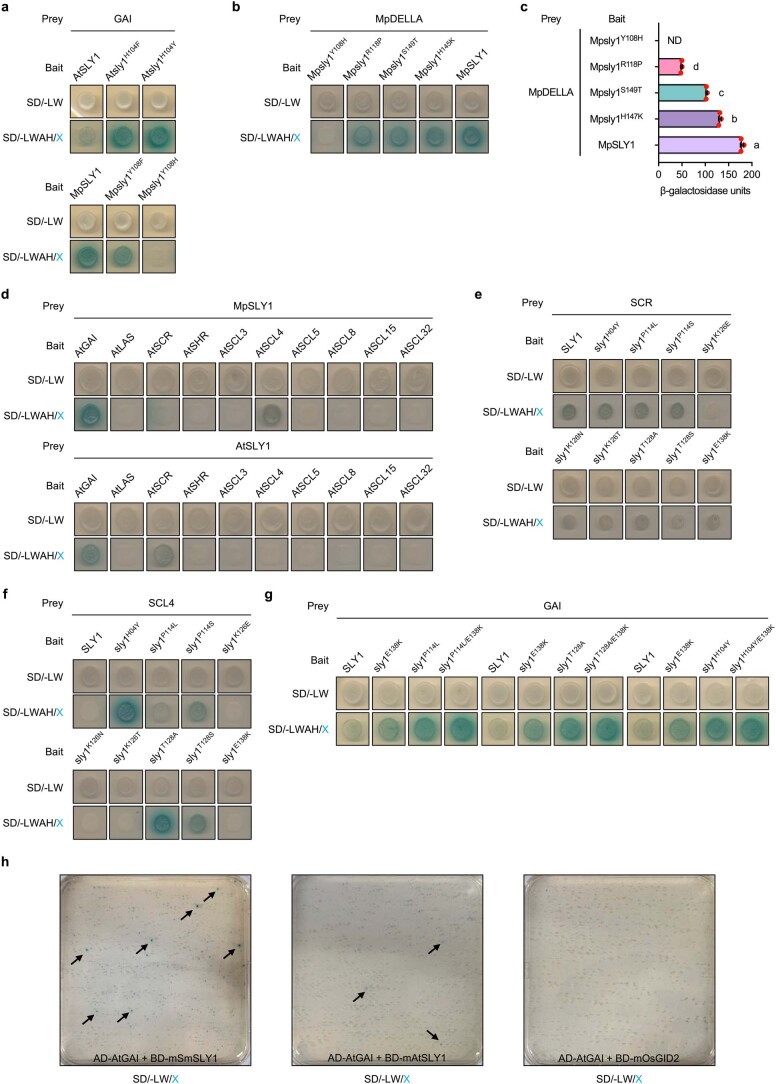
SLY1 reversion routes are evolutionarily constrained. **a**, Interactions of AtSLY1 and Atsly1 variants or MpSLY1 and Mpsly1 variants with GAI. **b**, Interactions of MpSLY1 and Mpsly1 variants with MpDELLA. **c**, Mean yeast-2-hybrid interaction strengths, Mpsly1 variants as in c, note gradient of increasing interaction strength ranging from Mpsly1^Y108H^ to MpSLY1. ND = not detected; red dots indicate individual values (n = 3), error bars indicate standard deviation, different letters (a-d) indicate significant difference (one-way ANOVA with Tukey’s test). **d**, Interactions of MpSLY1 and AtSLY1 with 10 representative *Arabidopsis* GRAS family proteins. **e**, Interactions of AtSLY1 and Atsly1 variants with AtSCR. **f**, Interactions of AtSLY1 and Atsly1 variants with AtSCL4. **g**, Interactions of AtSLY1 and Atsly1 variants with GAI. **h**, Example mutant screen plates, mSmSLY1 (left), mAtSLY1 (centre), mOsGID2 (right). Positive candidates (blue colonies) are indicated with arrows. Source data

The H104Y charged to hydrophobic side-chain substitution has the greatest observed effect on AtSLY1 A affinity (Fig. 2b f). We found that Mpsly1^Y108H^ (MpSLY1 108 is equivalent to AtSLY1 104) abolished MpSLY1-MpDELLA interactions (Fig. 5b), suggesting that loss of Y108 (substitution with H) contributed to the reduced angiosperm SLY1 A affinity. Conversely, gid2^H145Y^ (GID2 145 is equivalent to AtSLY1 104) exhibited weakly but detectably enhanced GID2 A affinity (for SLR1; Fig. 5b; the weak effect is probably because OsGID2 is more divergent, see below; Fig. 4a and Extended Data Fig. 5). Intriguingly, F (also hydrophobic side chain) occupies the SmSLY1 equivalent position and, while Atsly1^H104F^ displays enhanced GAI affinity (enhanced similarly to Atsly1^H104Y^), Mpsly1^Y108F^ exhibits reduced interaction (compared with MpSLY1; Fig. 5c and Extended Data Fig. 8a). We conclude that Y > H and Y > F substitutions at what became AtSLY1 position 104 (possibly by altering internal SLY1 GGF and LSL domain structural relations), contributed to the evolution of A’ specificity, and that Atsly1^H104Y^ is indeed evolutionarily revertant.

Systematic replacement of MpSLY1 residues (in equivalent positions) with the AtSLY1 residues substituted in the remaining Atsly1 variants (P114, K126 and T128, but not E138 because the MpSLY1 equivalent is also E) revealed all resultant variants (Mpsly1^R118P^, Mpsly1^H147K^ and Mpsly1^S149T^) to exhibit reduced MpDELLA affinity (Extended Data Fig. 8b,c); this suggests that the respective evolutionary substitutions (such as at the H104 equivalent) all contributed to evolutionary decline in A affinity. Further experiments testing selected *Arabidopsis* non-DELLA GRAS proteins showed that AtSLY1 interacts with AtSCR alone (Extended Data Fig. 8d). Conversely, MpSLY1 interacts with AtSCL4 alone (Extended Data Fig. 8d). Next, while no Atsly1 variants exhibited detectably enhanced AtSCR interactions, several of them (Atsly1^H104Y^, Atsly1^P114L^, Atsly1^P114S^, Atsly1^T128A^ and Atsly1^T128S^) exhibited novel AtSCL4 interactions (Extended Data Fig. 8e,f). Thus, these variants exhibit a broadened affinity mimicking that of MpSLY1, again suggesting them to be evolutionarily revertant.

### Evolutionary constraints on SLY1 reversion

The partially increased A affinity of gid2^H145Y^ (Fig. 5b) suggests the sometimes-limited effects of single-residue substitutions. Accordingly, pairwise AtSLY1 substitutions additively increase A affinity (Fig. 5d and Extended Data Fig. 8g). For example, an H104Y/E138K combination enhances affinity close to the ‘maximum’ MpSLY1-MpDELLA affinity (Figs. 4c and 5d), indicating that evolutionary enhancement of A’ specificity (reducing A affinity) probably involved multiple substitutions. Further experiments showed that evolution of SLY1 away from A + A’ affinity increasingly ‘locked’ A’ specificity (reduced the number of positions where single substitutions could restore A affinity; Fig. 5e and Extended Data Fig. 8h). Multiple SmSLY1 (least diverged; Fig. 4a) sites (14), fewer AtSLY1 (medium divergence; Fig. 4a) sites (5) and very few OsGID2 (highly diverged; Fig. 4a) sites (3) were targeted (with some targeted sites in equivalent positions; for example, SmSLY1^S129^, AtSLY1^T128^ and OsGID2^S180^; Fig. 5e). We also replicated 9 Atsly1 substitutions (Fig. 2a) at equivalent GID2 positions, finding that only H145Y (as above, interaction was too weak for detection in the screen) and S180A (recovered in the screen) enhanced SLR1 interactions (Fig. 5f). However, combining H145Y and R155L (GID2 R155 is equivalent to AtSLY1 P114), or R155L and Q190K (GID2 Q190 is equivalent to AtSLY1 E138) significantly increased gid2^H145Y/R155L^ and gid2^R155L/Q190K^ SLR1 interactions (Fig. 5f). We conclude that GID2 is so locked into A’ specificity that reverting it typically requires multiple substitutions. Conversely, because AtSLY1 and SmSLY1 are less locked, single substitutions can detectably increase A affinity.

## Discussion

DELLAs regulate plant biology via interaction with multiple transcription factors^2^. Post-translational modifications (for example, phosphorylation, SUMOylation, glycosylation) influence these interactions, thus modulating DELLA activity^30^. In contrast, how the DELLA A to A’ transition increases DELLA–SCF^SLY1/GID2^ interactions, and how A’ specificity evolved, was hitherto little understood. Collectively, our findings reveal progressive refinement of ancestral SLY1 dual A + A’ affinity towards A’ specificity during land-plant evolution.

DELLA function predated GA signalling^2,31^. Furthermore, the N-terminal DELLA domain probably had an initial transcriptional transactivation function^19^, implying its subsequent recruitment for growth-regulatory GA–GID1–DELLA complex formation. Because such complex formation can directly influence DELLA function (via a mechanism not involving destruction^2^), recruitment of SLY1-mediated A vs A’ differential proteasome-dependent DELLA destruction may have been a further step in the evolution of GA–GID1–DELLA signalling. While our observations suggest continued refinement of A’ specificity post vascularization, this likelihood would benefit from further investigation. Alternatively, because SLY1 is present in the bryophyte lineage^19^ and because MpSLY1 binds DELLA, it is possible that SLY-mediated DELLA regulation was established before GID1 recruitment^22^.

Interestingly, enhanced A affinity Atsly1 variants frequently revert evolutionary substitutions that were probably causal of A’ refinement. For example, Atsly^H104Y^ restores the Y of basal SLY1s. This predictability contrasts with findings that narrowing of initial animal dual B-cell lymphoma-2 (BCL-2) affinity for BID1 and NOXA partners to specificity for BID1 was achieved experimentally via substitutions at sites often not evolutionarily targeted^32^. Perhaps our study reveals greater predictability because it concerns evolving affinities for distinct (A/A’) conformations of the same protein (rather than different BID1 and NOXA proteins). Progressive A’ refinement also incurs ‘locking’. GID2 has so many substitutions (for example, relative to MpSLY1) that it is effectively almost entirely locked into A’ specificity: relatively few single substitutions enable reversion to dual A + A’ affinity. In a comparable study, negative interaction ‘locking’ elements have been shown to maintain insulation between two paralogous bacterial toxin–antitoxin systems^33^.

Despite recent advances in understanding of PPI^34^, relatively few studies address the evolution of PPI specificity^32,35–41^. While the extent to which specificity enhancement is a general driver of PPI evolution is debated^42^, our study suggests the importance of affinity narrowing and provides a specific example. First, a change in SLY1 core conformation (Y to H substitution at the buried site equivalent to AtSLY1 104) sometime before angiosperm divergence reduced A (vs A’) affinity (perhaps by a change in LSL accessibility). Second, mutation accumulation during angiosperm evolution (for example, at sites AtSLY1 126/138) reduced electrostatic interactions with A at the interaction interface. The resultant trend towards SLY1/GID2 A’ specificity was probably driven by selective advantage: consequent GA regulation enhanced the flexibility of adaptational plant growth control in response to environmental variables.

## Methods

### Plant materials and growth conditions

*Arabidopsis thaliana* seeds were sterilized with 75% ethanol for 10 min and germinated on half-strength Murashige and Skoog (MS, Sigma-Aldrich, M5519) salt medium (pH 5.8) containing 0.5% sucrose, 0.5 g l^−1^ 2-morpholinoethanesulfonic acid (MES) and 1% agar at 22 °C in a 16 h light/8 h dark photoperiod (irradiance 120 μmol m^−2^ s^−1^). Seedlings (10-day-old) were transplanted to soil (ICL Levington advanced F2 compost) and grown in controlled environment rooms (CERs) in the same environmental conditions as above. *M. polymorpha* accession Takaragaike-1 (Tak-1; male) was cultured on half-strength MS medium (pH 5.6) containing 0.5% sucrose, 0.5 g l^−1^ MES and 0.8% agar in the same growth conditions. *S. moellendorffii* plants were kindly provided by the University of Bristol Botanic Garden and maintained at 24 °C in a 16 h light/8 h dark photoperiod (irradiance 90 μmol m^−2^ s^−1^) in plant growth incubators (Sanyo, MLR 351). Seeds of *O. sativa* subspecies *japonica* (Nipponbare variety) were peeled and sterilized in 70% ethanol for 30 s, followed by 15 ml of 10% sodium hypochlorite (NaClO) for 30 min. The seeds were then thoroughly rinsed with distilled water and stratified at 37 °C in the dark for 3–7 d. Following germination, rice seedlings were transferred to 1.5 l hydroponic devices containing 1 l of half-strength MS liquid media supplemented with vitamins (Duchefa Biochemie, M0222) in plant growth incubators (Sanyo, MLR 351) set at 22 °C in a 16 h light/8 h dark photoperiod (irradiance 200 μmol m^2^ s^−1^). Nutrient solution was replaced every 3 d until the tissue was ready to harvest.

### *Arabidopsis* seed mutagenesis and mutant screen

Approximately 50,000 *Arabidopsis gai* progenitor seeds (La-er background) were mutagenized by incubating in 0.2% ethyl methane sulfonate (EMS, Sigma-Aldrich, M0880) for 15 h, followed by 10 washes with distilled water. The seeds (M_1_) were then separated into batches of ~2,000 and sown on soil. The M_1_ plants were allowed to self-pollinate and the resultant M_2_ seeds were collected for genetic screens. Mutants that were visibly taller than the *gai* progenitor were selected for heritability and segregation tests and taken to the subsequent M_3_ generation. Leaf material from homozygous mutant populations was collected for DNA extraction and *gai* was sequenced to eliminate *gai* loss-of-function mutations (that is, *gai-d* mutations^1,24^). For the remainder, *SLY1* gene sequencing identified *sly1* mutations conferring the mutant phenotype. Primers used to amplify *gai* and *SLY1* from genomic DNA for Sanger sequencing are listed in Supplementary Table 3.

### Leaf chlorophyll measurement

Leaf chlorophyll content was measured using a SPAD-502 metre (Konica-Minolta) as previously described^43^. Absolute chlorophyll concentration in nmol mg^−1^ fresh weight was calculated using the previously derived equation:

1 $${y}=0.0007{x}^{2}+0.0230x+0.0544$$

where *y* is the absolute chlorophyll concentration and $\,{x}$ is the SPAD metre reading^43^.

### *Arabidopsis* transformation

DNA fragments consisting of the promoter (~2 kbp upstream of the transcription start site) and genomic DNA sequences of *SLY1* and *sly1*^*G84D*^ were amplified from *gai* and *gai* *gar2-2* *Arabidopsis* plants, respectively, then cloned into pCAMBIA2300 to make *pSLY1*::*SLY1* and *pSLY1*::*sly1*^*G84D*^ constructs. *pSLY1*::*sly1*^*H104Y*^ and *pSLY1*::*sly1*^*T128A*^ were generated using Q5 site-directed mutagenesis (NEB, E0554S) of *pSLY1*::*SLY1*. To make the *35S::HA*-*MpSLY1* overexpression construct, the coding sequence of *MpSLY1* was amplified from complementary DNA (cDNA) and cloned into the pEarlyGate201 vector. All constructs were transformed into *gai* (Col-0 background) using the *Agrobacterium* (GV3101 strain)-mediated floral dip method^44^. Relevant primer sequences are listed in Supplementary Table 3.

### RNA isolation, cDNA synthesis and quantitative PCR with reverse transcription (RT–qPCR) analysis

Total RNA was extracted from ~100 mg plant material using TRIzol reagent (ThermoFisher, 15596026) and treated with the DNA-free DNA removal kit (ThermoFisher, AM1906) following manufacturer instructions. Full-length cDNA was subsequently reverse transcribed using SuperScript IV reverse transcriptase (ThermoFisher, 18090010) before being used for RT–qPCR on an Applied Biosystem StepOnePlus real-time PCR system (Thermo Scientific) using the qPCRBIO SyGreen Mix Hi-Rox reagent (PCR Biosystems, PB20.12). RT–qPCR assays included three biological replicates, and the results were analysed using the StepOnePlus software v.2.3 and Microsoft Office Excel v.16.71. The *Arabidopsis Actin2* gene (At3g18780) was used as internal control. Primers used for RT–qPCR are listed in Supplementary Table 3.

### Mutagenic EP-PCR

The EP-PCR reaction mixture consisted of the following: 10 mM Tris-HCl (pH 8.3), 50 mM KCl, 7 mM MgCl_2_, 0.5 mM MnCl_2_, 1 mM dCTP, 1 mM dTTP, 0.2 mM dATP, 0.2 mM dGTP, 2 μM 5’ primer, 2 μM 3’ primer, 0.05 U μl^−1^ *Taq* DNA polymerase, 20 pg μl^−1^ DNA template and if necessary, 3% dimethyl sulfoxide. Thirty-five cycles of PCR were performed at a *T*_m_ of 55 °C. The resultant enhancement of the natural error rate of the *Taq* polymerase was due to the elevated MgCl_2_ concentration, the presence of MnCl_2_ (which stabilizes non-complementary nucleotide pairing) and an uneven ratio of nucleotides in the reaction. The resultant mutagenized DNA libraries were gel purified with the QIAquick gel extraction kit (QIAGEN, 28704), then cloned into pGBKT7 vector using In-Fusion Snap Assembly (TaKaRa, 638947). Primers used for EP-PCR are listed in Supplementary Table 3. EP-PCR enables semi-random mutation generation and is not necessarily a reliable proxy for the multiple mechanisms via which mutations are generated during biological evolution.

### Yeast 2-hybrid assay and mutant screen

Various *SLY1* and *DELLA* coding sequences were amplified from cDNA and cloned into vectors pGBKT7 and pGADT7 to generate bait (pGBKT7-SLY1) and prey (pGADT7-DELLA) constructs, respectively. Bait and prey constructs (100 ng) were co-transformed into yeast strain AH109 (TaKaRa) and selected on the synthetic defined (SD) yeast leucine and tryptophan dropout medium (SD/-LW) for 3 d at 30 °C. At least four colonies from each transformation were selected at random and resuspended in 50 μl of 0.9% NaCl, 5 μl of which was spotted onto the SD/-LW (for loading control) and the leucine, tryptophan, adenine and histidine dropout medium supplemented with 40 μg ml^−1^ X-α-Gal (SD/-LWAH/X, for assessing interaction strength). The plates were then incubated for 3–5 d at 30 °C. Relevant primer sequences are listed in Supplementary Table 3.

For the yeast 2-hybrid mutant screen, AH109 yeast cells were first transformed with pGADT7-GAI and maintained on leucine dropout medium (SD/-L), followed by transformation with 100–200 ng of bait vectors containing EP-PCR-mutagenized *SLY1/GID2* libraries. For each screen, at least 3,000 colonies were plated on SD/-LW medium supplemented with 40 μg ml^−1^ X-α-Gal (SD/-LW/X). Colonies turning blue after 3–4 d were selected and cultured overnight in liquid tryptophan dropout medium (SD/-W) for plasmid extraction with the Zymoprep yeast plasmid miniprep kit (Zymo Research, D2001). *SLY1/GID2* DNA was then PCR-amplified from the extracted plasmid for Sanger sequencing (Source BioScience), thus enabling detection of EP-PCR-generated mutations potentially responsible for the selected enhanced bait–prey interaction. Likely candidate mutations were first identified as those recovered at least three times from the screens. These candidate mutations were next reconstructed from the original pGBKT7-SLY1/GID2 vectors using Q5 site-directed mutagenesis (NEB, E0554S) to remove additional potentially confounding EP-PCR-generated mutations, and confirmed genuine by performing yeast 2-hybrid assays with their respective DELLA partners (SmDELLA, AtGAI or OsSLR1). All screens were performed at least four times using independently mutagenized libraries to avoid repetitively selecting clonal candidates.

### β-galactosidase quantification of yeast 2-hybrid interactions

Yeast 2-hybrid quantitative assays were performed with strain Y187 (in liquid culture) using chlorophenol red β-d-galactopyranoside (CPRG, Roche, 11379119103) as substrate. For each interaction pair, three colonies were cultured overnight in SD/-LW liquid before being diluted 5-fold in liquid rich medium, and grown further until the optical density (OD)_600_ was within the 0.5–0.8 range. The culture (1.5 ml) was then pelleted and resuspended in 300 μl buffer 1 (2.38 g 4-(2-hydroxyethyl)-1-piperazineethanesulfonic acid (HEPES), 0.9 g NaCl, 0.065 g l-aspartate, 1 g BSA and 50 μl Tween-20 in 100 ml solution, pH adjusted to 7.25–7.30, filter sterilized). The cell suspension was next divided into three 100 μl aliquots and cells were broken open by repetitively (at least three times) freezing the culture in liquid nitrogen, followed by immediate rapid thawing in a 37 °C water bath. Buffer 2 (0.7 ml) (2.23 mM CPRG in buffer 1, filter sterilized) was then added to start the reaction. The reaction was stopped when the colour of the sample turned orange/red by adding 0.5 ml 3 mM ZnCl_2_. Cell debris was removed by spinning and the OD_578_ of the supernatant was recorded using the Evolution 260 BIO UV-visible spectrophotometer with INSIGHT2 software. β-galactosidase activity (units) was calculated using the following equation:

2 $${y}=1,000\times {{\rm{OD}}}_{578}/({t}\times {V}\times {{\rm{OD}}}_{600})$$

where *y* is the β-galactosidase unit; *t* is the elapsed time (in minutes) of incubation; *V* is 0.1 × concentration factor (in this case *V* = 0.5). An interaction was deemed ‘not detected’ (ND) if the OD_578_ was <0.01 after 3 h of colour development.

### Yeast 3-hybrid assay

*SLY1* and *GID1* coding sequences were amplified from cDNA and cloned into the pBridge vector (TaKaRa), with *SLY1* fused with the GAL4 DNA-binding domain and *GID1* fused with the Met promoter. Prey and pBridge vectors were co-transformed into strain AH109 that had previously been streaked three times on methionine dropout media (SD/-M). Transformed colonies were selected on methionine, leucine and tryptophan dropout medium (SD/-LWM) 5–7 d after transformation. At least five colonies for each interaction pair were selected at random and resuspended in 50 μl 0.9% NaCl, 5 μl of which was spotted onto the SD/-LWM (for the loading control), SD/-LWAHM medium supplemented with X-α-Gal (SD/-LWAHM/X) and SD/-LWAHM/X medium supplemented with 0.1 mM GA_3_. The plates were then incubated for 3–5 d at 30 °C. Relevant primer sequences are listed in Supplementary Table 3.

### Western blot analysis

Total protein was extracted from ~100 mg of plant material using extraction buffer containing 50 mM Tris-HCl (pH 7.5), 150 mM NaCl, 0.1% NP-40 detergent, 10% glycerol, 1 mM dithiothreitol and protease inhibitor cocktail (Roche, 11697498001). Yeast protein was extracted from liquid overnight culture using YeastBuster (Merck, 71186) supplemented with Tris(hydroxypropyl)phosphine solution (Merck, 71194) and protease inhibitor cocktail (Roche, 11697498001) following manufacturer instructions. Protein samples were heated at 70 °C for 10 min before being subjected to sodium dodecyl sulfate–polyacrylamide gel electrophoresis (SDS–PAGE) and transferred to a nitrocellulose membrane (VWR, PIER88013). The membrane was stained in Ponceau S solution (Sigma-Aldrich, P7170), which was subsequently washed off with 0.1 M NaOH before blocking. GAI, SLY1 and Actin proteins in plant extracts were detected using AF2 (1:5,000)^14^, anti-SLY1 (Agrisera, AS13 2638, 1:5,000) and anti-Actin (Agrisera, AS13 2640, 1:5,000), respectively. RPT5 (regulatory particle triple-A ATPase 5), HA-tagged GAI and Myc-tagged SLY1 proteins in yeast extracts were detected using anti-RPT5 (abcam, ab22676, 1:10,000), anti-HA (MBL Life science, M180-7, 1:5,000) and anti-Myc (MBL Life science, M192-7, 1:5,000), respectively. The membranes were visualized on an iBright FL1500 imaging system (ThermoFisher). Band intensity was quantified using gel analysis methods (ImageJ).

### Protein purification and pull-down assay

To obtain recombinant His-tagged GAI/gai and MBP-tagged SLY1/sly1 proteins, the coding sequences of WT and mutant variants of *GAI* and *SLY1* were amplified from cDNA or pGBKT7 bait vectors recovered from yeast 2-hybrid screens and cloned into pCold-TF (TaKaRa) and pMAL-c2X (NEB), respectively. The coding sequence of *GID1C* was amplified from cDNA and cloned into pGEX-4T-1 (GE Healthcare). These constructs were transformed into *E. coli* strain BL21 for protein expression. Recombinant His-GAI/gai, MBP-SLY1/sly1 and GST-GID1C proteins were purified using Ni-NTA agarose (QIAGEN, 30210), amylose resin (NEB, E8021S) and glutathione beads (Smart-Lifesciences, SA008100), respectively, according to manufacturer instructions. For the in vitro pull-down assay, 5 μg His-GAI/gai was incubated with Ni-NTA agarose (QIAGEN, 30210) for 3 h at 4 °C. After centrifugation, the supernatant was removed and 5 μg MBP-SLY1/sly1 was added to the pelleted beads. When required, an equal amount of GST-GID1C and 0.1 mM GA_3_ were added to the reaction. After a further 1 h incubation at 4 °C, the beads were washed five times before being resuspended in SDS loading buffer. The proteins were released into the solution by boiling for 5 min and detected through immunoblots using anti-His (Santa Cruz, SC-8036, 1:2,000), anti-MBP (NEB, E8032S, 1:10,000) and anti-GST (MBL, PM013-7, 1:5,000) antibodies. Relevant primer sequences are listed in Supplementary Table 3. Band intensity was quantified using gel analysis methods (ImageJ).

### Cell-free degradation assay

Total protein was extracted from 14-day-old *Arabidopsis* seedlings in lysis buffer (25 mM Tris-HCl (pH 7.5), 10 mM NaCl, 10 mM MgCl_2_, 4 mM phenylmethylsulfonyl fluoride, 5 mM dithiothreitol and 10 mM ATP). Protein concentration was determined using the Bradford protein assay (ThermoFisher, B6916) and adjusted to 5 μg μl^−1^. Plant extract (150 μl) was incubated with 100 ng of recombinant His-gai protein at room temperature, with samples taken at a series of timepoints. Proteins were detected through immunoblots using anti-His (Santa Cruz, SC-8036, 1:2,000) and anti-Actin (EASYBIO, BE0021, 1:5,000) antibodies. Band intensity was quantified using gel analysis methods (ImageJ).

### Phylogenetic analysis

Protein sequences of SLY1/GID2 orthologues from diverse land plants were obtained from a variety of sources including Phytozome^45^, PLAZA^46^, OneKP^47^ and FernBase^48^, using SmSLY1 and SmSLY2 as queries for BLASTP with an expected (*e*-value) threshold of 1 × 10^−20^. Multiple sequence alignment was performed using the T-Coffee alignment server^49^ and phylogenetic trees were constructed in MEGA11 (ref. ^50^) using the maximum-likelihood method and the Jones–Taylor–Thornton (JTT) matrix-based model.

### Protein structure predictions

Predicted SLY1 structures were obtained from the AlphaFold Protein Structure Database^25,26^ (Q9STX3), or predicted by RoseTTAFold^27^ or ESMFold^28^. The structure of the GAI-SLY1 complex was predicted by AlphaFold2-multimer^51^. All protein structures were visualized, analysed and annotated in PyMOL.

### Statistics and reproducibility

All statistical analyses (Student’s *t*-test, one-way analysis of variance (ANOVA) with Tukey’s test and chi-square test) were performed using GraphPad Prism 9. *P* < 0.05 was considered to indicate statistical significance. Exact *P* values are provided either in the figure legends, supplementary tables or source data. All western blot, pull-down and cell-free degradation assays were repeated three times with similar results.

### Reporting summary

Further information on research design is available in the Nature Portfolio Reporting Summary linked to this article.

### Supplementary information

Reporting Summary

Supplementary Tables Supplementary Table 1 Amino acid substitutions in Atsly1 variants selected in yeast-two-hybrid screens. Table 2 Chi-square analysis of frequencies of expected vs observed amino acid substitutions. Table 3 List of primers used in this study.

### Source data

Source Data Figs. 1–5 and Extended Data Figs. 1–3, 6 and 8 Statistical source data.

Source Data Figs. 1 and 2, and Extended Data Figs. 2 and 6. Unprocessed western blots.

## Data Availability

All data generated in this study are included in the main text and in the Supplementary Information. Structural models of AtSLY1 (UniProt ID: Q9STX3) and AtGAI (UniProt ID: Q9LQT8) were obtained from the AlphaFold database. All experimental materials constructed in this study are available from the corresponding author upon request. Source data are provided with this paper.
