# Supplementary material for: Evolution of a plant growth-regulatory protein interaction specificity
Source: Nat Plants. 2023 Oct 30;9(12):2059–70. doi: 10.1038/s41477-023-01556-0 (PMC10724065; doi:10.1038/s41477-023-01556-0)
Supplement: Supplementary file 1 — Reporting Summary [file 41477_2023_1556_MOESM1_ESM.pdf]

nature portfolio

Corresponding author(s): Nicholas P. Harberd

Last updated by author(s): Sep 20, 2023

Reporting Summary

Nature Portfolio wishes to improve the reproducibility of the work that we publish. This form provides structure for consistency and transparency in reporting. For further information on Nature Portfolio policies, see our Editorial Policies and the Editorial Policy Checklist.

Statistics

For all statistical analyses, confirm that the following items are present in the figure legend, table legend, main text, or Methods section.

n/a	Confirmed	
☐	☒	The exact sample size (*n*) for each experimental group/condition, given as a discrete number and unit of measurement
☐	☒	A statement on whether measurements were taken from distinct samples or whether the same sample was measured repeatedly
☐	☒	The statistical test(s) used AND whether they are one- or two-sided<br>*Only common tests should be described solely by name; describe more complex techniques in the Methods section.*
☒	☐	A description of all covariates tested
☐	☒	A description of any assumptions or corrections, such as tests of normality and adjustment for multiple comparisons
☐	☒	A full description of the statistical parameters including central tendency (e.g. means) or other basic estimates (e.g. regression coefficient) AND variation (e.g. standard deviation) or associated estimates of uncertainty (e.g. confidence intervals)
☐	☒	For null hypothesis testing, the test statistic (e.g. *F*, *t*, *r*) with confidence intervals, effect sizes, degrees of freedom and *P* value noted<br>*Give P values as exact values whenever suitable.*
☒	☐	For Bayesian analysis, information on the choice of priors and Markov chain Monte Carlo settings
☒	☐	For hierarchical and complex designs, identification of the appropriate level for tests and full reporting of outcomes
☒	☐	Estimates of effect sizes (e.g. Cohen's *d*, Pearson's *r*), indicating how they were calculated

Our web collection on statistics for biologists contains articles on many of the points above.

Software and code

Policy information about availability of computer code

Data collection	qPCR data were collected using the Applied Biosystem StepOnePlus Real-Time PCR System Software v2.3; Western blot data were collected using the iBright FL1500 Imaging System Firmware 1.7.0; Absorbance data were collected using the Evolution 260 BIO UV-Visible Spectrophotometer with INSIGHT2 software.
Data analysis	qPCR data were analysed using the StepOnePlus Software v2.3 and Microsoft Office Excel v16.71; Phylogeny was analysed using MEGA11; Protein structures were analysed using PyMOL v2.5.2; Statistical analyses were conducted and figures generated in GraphPad Prism v9.5.1. Band intensities for Western Blot analyses were quantified by ImageJ (bundled with Zulu OpenJDK) 13.0.6.

For manuscripts utilizing custom algorithms or software that are central to the research but not yet described in published literature, software must be made available to editors and reviewers. We strongly encourage code deposition in a community repository (e.g. GitHub). See the Nature Portfolio guidelines for submitting code & software for further information.

Data

Policy information about availability of data

All manuscripts must include a data availability statement. This statement should provide the following information, where applicable:

- Accession codes, unique identifiers, or web links for publicly available datasets
- A description of any restrictions on data availability
- For clinical datasets or third party data, please ensure that the statement adheres to our policy

All data generated in this study are included in the main text and supplementary information of this article. Structural models of AtSLY1 (UniProt ID: Q9STX3) and AtGAI (UniProt ID: Q9LQT8) were obtained from the AlphaFold database. All experimental materials constructed in this study are available from the corresponding author upon request. Source data are provided with this paper.

Human research participants

Policy information about studies involving human research participants and Sex and Gender in Research.

Reporting on sex and gender	N.A.
Population characteristics	N.A.
Recruitment	N.A.
Ethics oversight	N.A.

Note that full information on the approval of the study protocol must also be provided in the manuscript.

Field-specific reporting

Please select the one below that is the best fit for your research. If you are not sure, read the appropriate sections before making your selection.

☒ Life sciences ☐ Behavioural & social sciences ☐ Ecological, evolutionary & environmental sciences

For a reference copy of the document with all sections, see nature.com/documents/nr-reporting-summary-flat.pdf

Life sciences study design

All studies must disclose on these points even when the disclosure is negative.

Sample size	Details of sample size and statistical analysis were described in Methods or relevant figure legends. No statistical methods were used to predetermine sample size. Sample sizes were determined based on previous publications performing similar experiments. At least three biological samples were analysed in all experiments in this manuscript to ensure sufficient sample sizes were included. Sample sizes were chosen on the basis of preliminary experimental trials for reproducibility of the assays, or previous publications in the field (e.g. Li et al., 2018 Nature; Wu et al., 2020 Science).
Data exclusions	No data were excluded from the analyses.
Replication	All experiments were repeated at least three times. Ten samples were analysed for plant phenotypic analyses. All reported results were reproducible and consistent.
Randomization	Plant samples were selected randomly for phenotypic, gene expression and protein analyses. Transformed plants were randomly selected for further analyses. Yeast colonies were selected randomly for qualitative and quantitative interaction analyses. No selection criteria were applied prior to sample allocation into experimental groups in this study.
Blinding	Pull-down and cell-free degradation assays were performed and analysed by authors without previous knowledge of the sample identities. For the other experiments, blinding was not applicable as the author who performed these experiments also analysed the results.

Reporting for specific materials, systems and methods

We require information from authors about some types of materials, experimental systems and methods used in many studies. Here, indicate whether each material, system or method listed is relevant to your study. If you are not sure if a list item applies to your research, read the appropriate section before selecting a response.

Materials & experimental systems

n/a	Involved in the study
☐	☒ Antibodies
☒	☐ Eukaryotic cell lines
☒	☐ Palaeontology and archaeology
☒	☐ Animals and other organisms
☒	☐ Clinical data
☒	☐ Dual use research of concern

Methods

n/a	Involved in the study
☒	☐ ChIP-seq
☒	☐ Flow cytometry
☒	☐ MRI-based neuroimaging

Antibodies

Antibodies used

anti-Actin (polyclonal): Agrisera; AS13 2640; Lot No. 2010; 1:5000
AF2/anti-GAI (monoclonal): 1:5000
anti-GID2/SLY1 (polyclonal): Agrisera; AS13 2638; Lot No. 1404; 1:5000
anti-RPT5/TBP-1 (polyclonal): abcam; ab22676; Lot No. GR3191288-1; 1:10000
anti-HA-tag mAb-HRP-DirecT (monoclonal): MBL; M180-7; Lot No. 008; 1:5000
anti-Myc-tag mAb-HRP-DirecT (monoclonal): MBL; M192-7; Lot No. 009; 1:5000
anti-His (monoclonal): Santa Cruz; sc-8036; 1:2000
anti-MBP (monoclonal): NEB; E8032S; 1:10000
anti-GST (polyclonal): MBL; PM013-7; Lot No. 010; 1:5000
anti-ACTIN (monoclonal): EASYBIO; BE0021; 1:5000

Validation

Validation statements and experiments can be obtained from the following websites:
anti-Actin: https://www.agrisera.com/en/artiklar/act-actin.html
anti-GAI (AF2): validated in the following publication: Sun, X. et al. N-terminal domains of DELLA proteins are intrinsically unstructured in the absence of interaction with GID1/gibberellic acid receptors. J Biol Chem 285, 11557-11571 (2010). https://doi.org:10.1074/jbc.M109.027011
anti-GID2/SLY1: https://www.agrisera.com/en/artiklar/gid2-f-box-protein-gid2-sleepy1-2.html
anti-RPT5/TBP-1: https://www.abcam.com/products/primary-antibodies/rpt5tbp-1-antibody-ab22676.html
anti-HA-tag mAb-HRP-DirecT: https://www.mblintl.com/products/m180-7/
anti-Myc-tag mAb-HRP-DirecT: https://www.mblintl.com/products/m192-7/
anti-His: https://www.scbt.com/p/his-probe-antibody-h-3
anti-MBP: https://www.neb.uk.com/products/neb-catalogue/protein-analysis,-exp-purification/anti-mbp-monoclonal-antibody
anti-GST: https://www.mblintl.com/products/pm013-7/
anti-ACTIN: http://www.bioeasytech.com/product/2377.html
