# Supplementary material for: Evolution of a plant growth-regulatory protein interaction specificity
Source: Nat Plants. 2023 Oct 30;9(12):2059–70. doi: 10.1038/s41477-023-01556-0 (PMC10724065; doi:10.1038/s41477-023-01556-0)

Fig. 1f

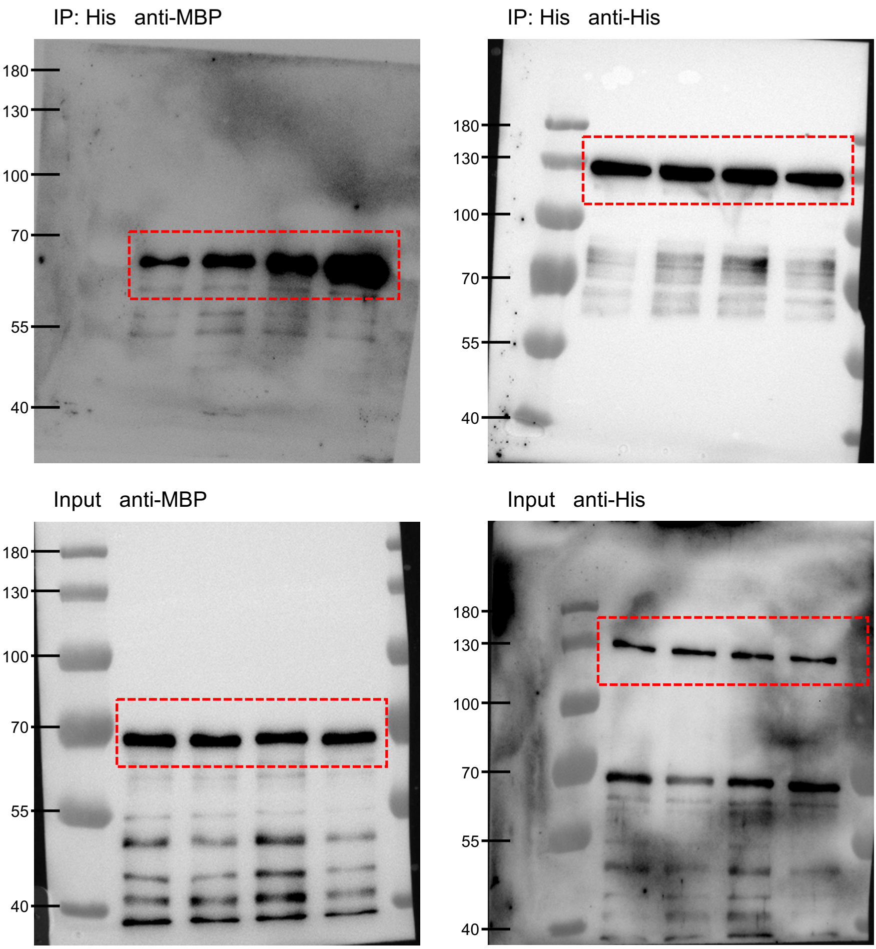
IP: His anti-MBP
IP: His anti-His
Input anti-MBP
Input anti-His
180
130
100
70
55
40

Fig. 1g

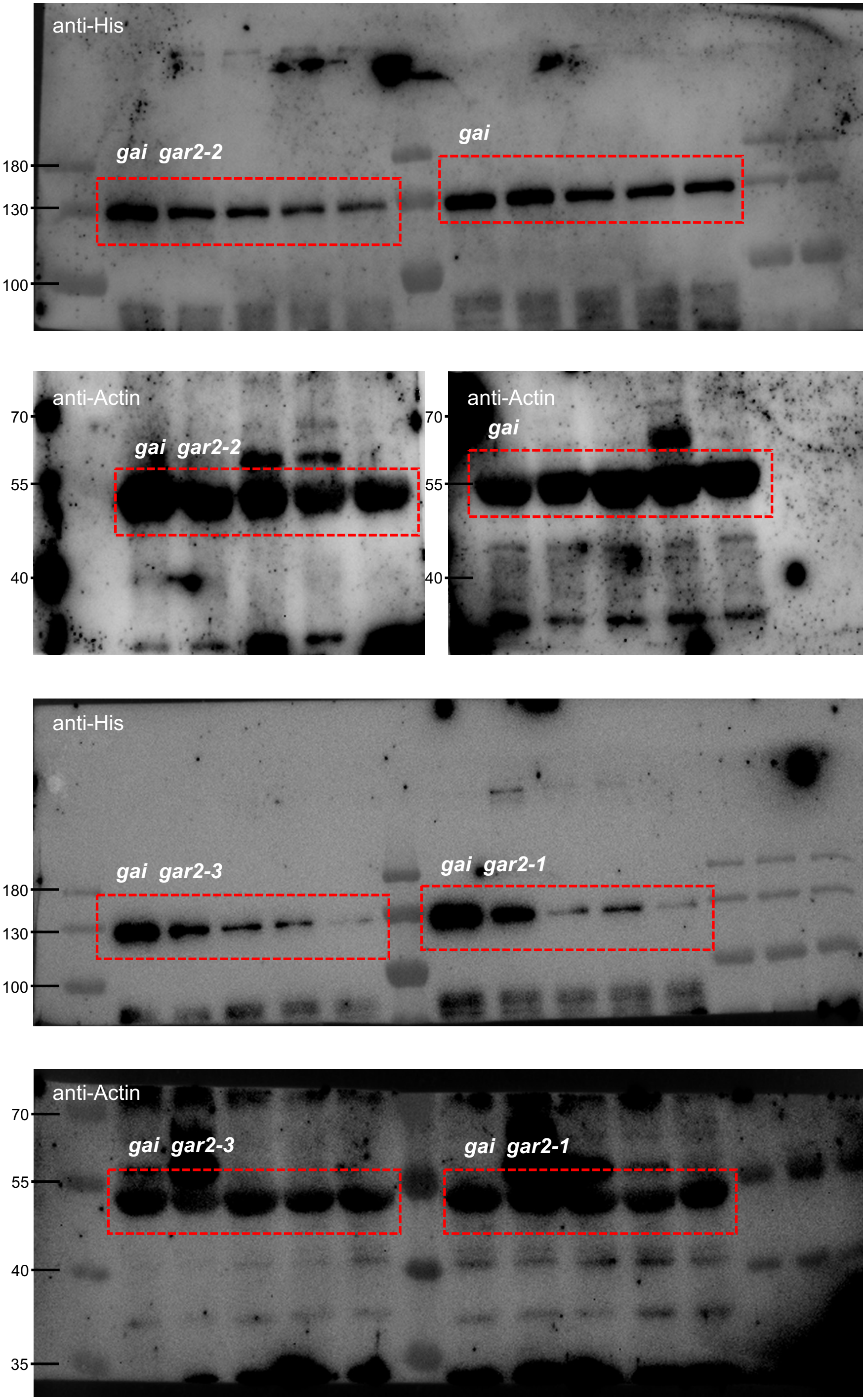
anti-His
gai gar2-2
gai
180
130
100
anti-Actin
gai gar2-2
70
55
40
anti-Actin
gai
70
55
40
anti-His
gai gar2-3
gai gar2-1
180
130
100
anti-Actin
gai gar2-3
gai gar2-1
70
55
40
35

Fig. 1h

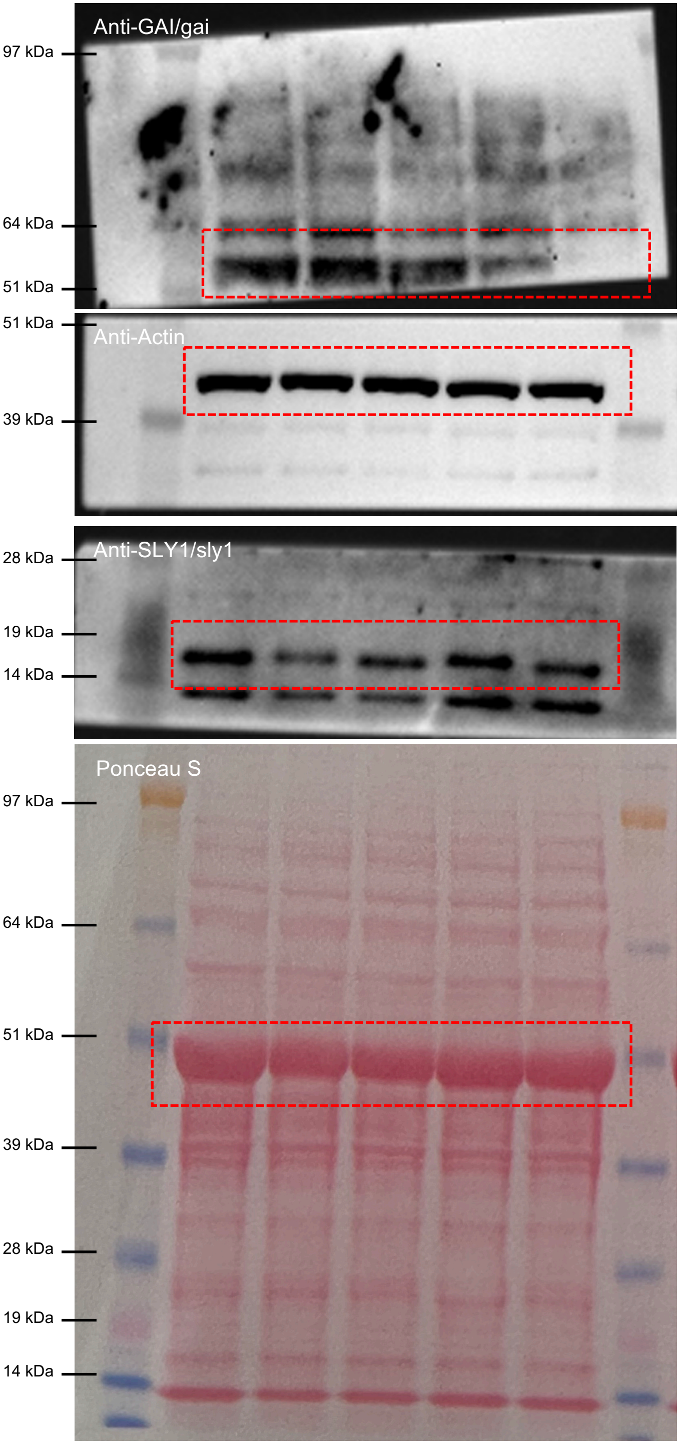
Anti-GAI/gai
97 kDa
64 kDa
51 kDa
51 kDa
Anti-Actin
39 kDa
Anti-SLY1/sly1
28 kDa
19 kDa
14 kDa
Ponceau S
97 kDa
64 kDa
51 kDa
39 kDa
28 kDa
19 kDa
14 kDa

Fig. 2c

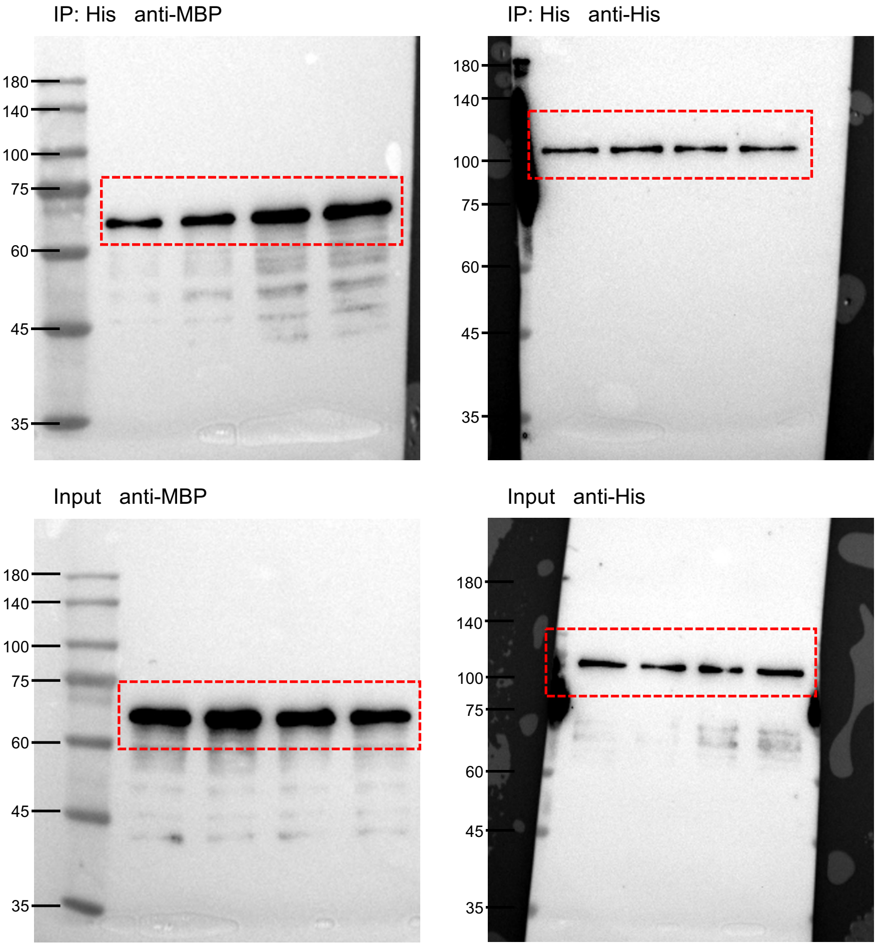
IP: His anti-MBP
180
140
100
75
60
45
35
IP: His anti-His
180
140
100
75
60
45
35
Input anti-MBP
180
140
100
75
60
45
35
Input anti-His
180
140
100
75
60
45
35

Fig. 2h

IP: His anti-MBP

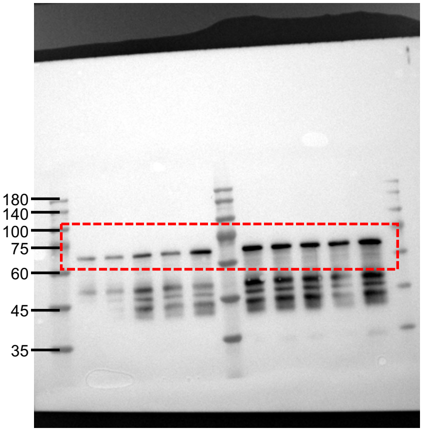

IP: His anti-GST

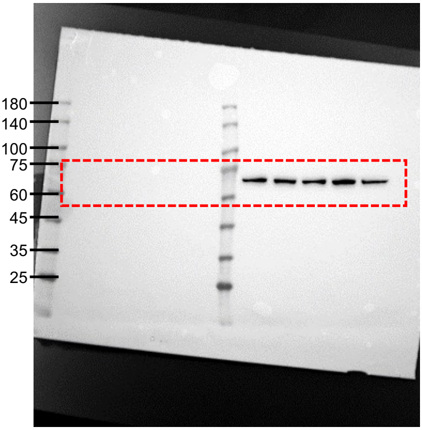

IP: His anti-His

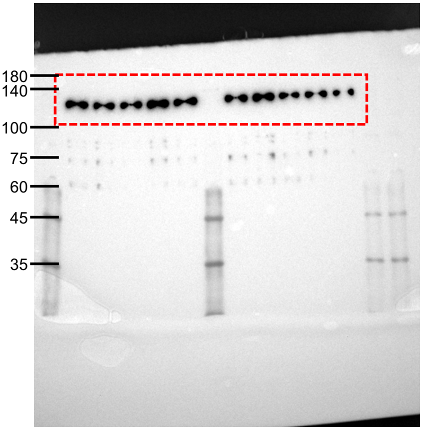

Input anti-MBP

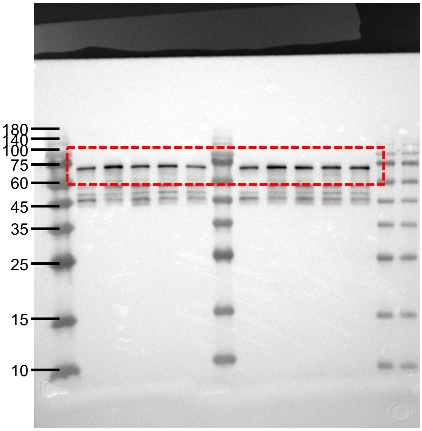

Input anti-GST

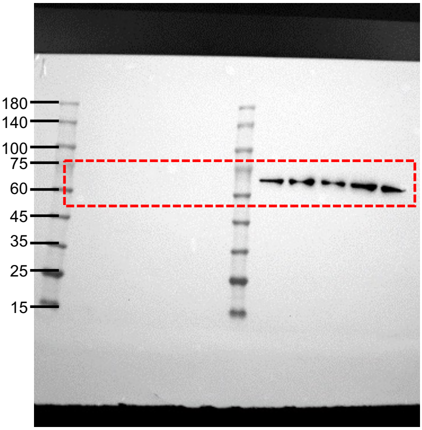

Input anti-His

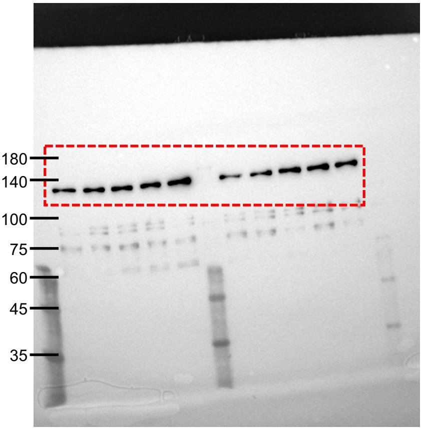

Extended Data Fig. 2d

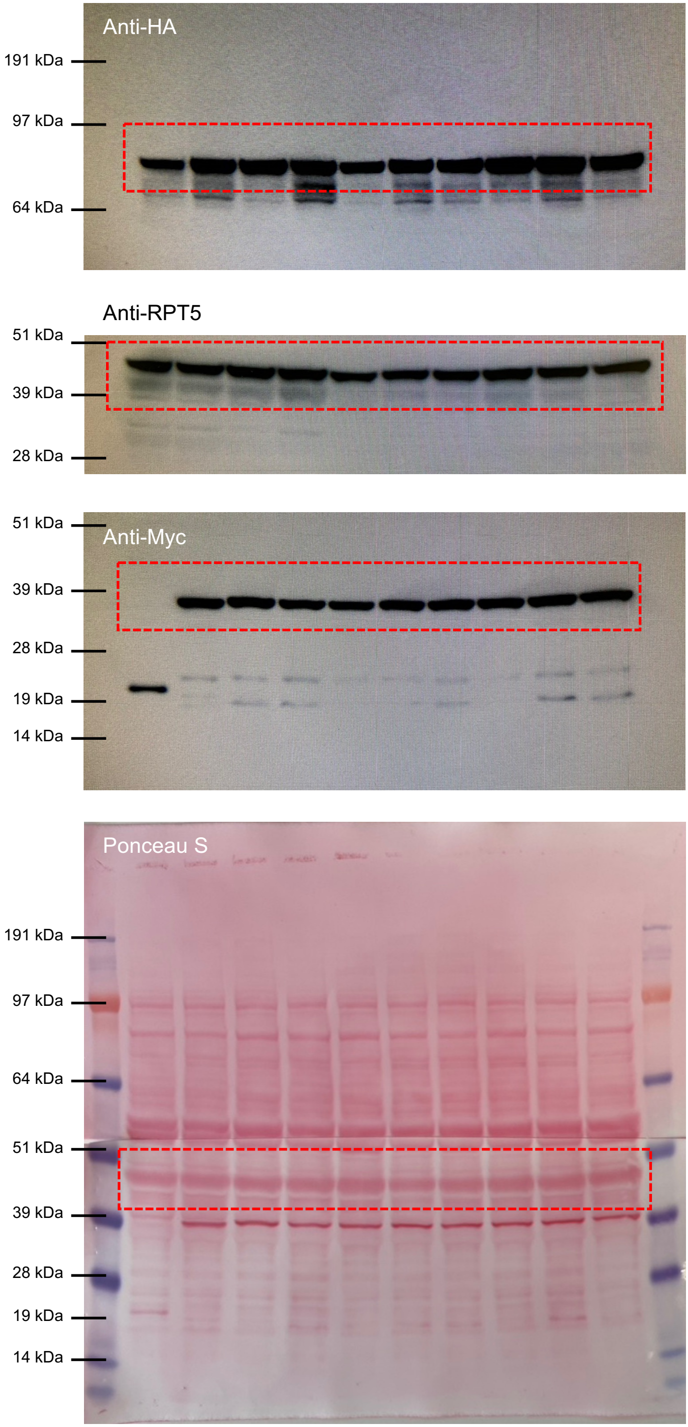

Extended Data Fig. 2h

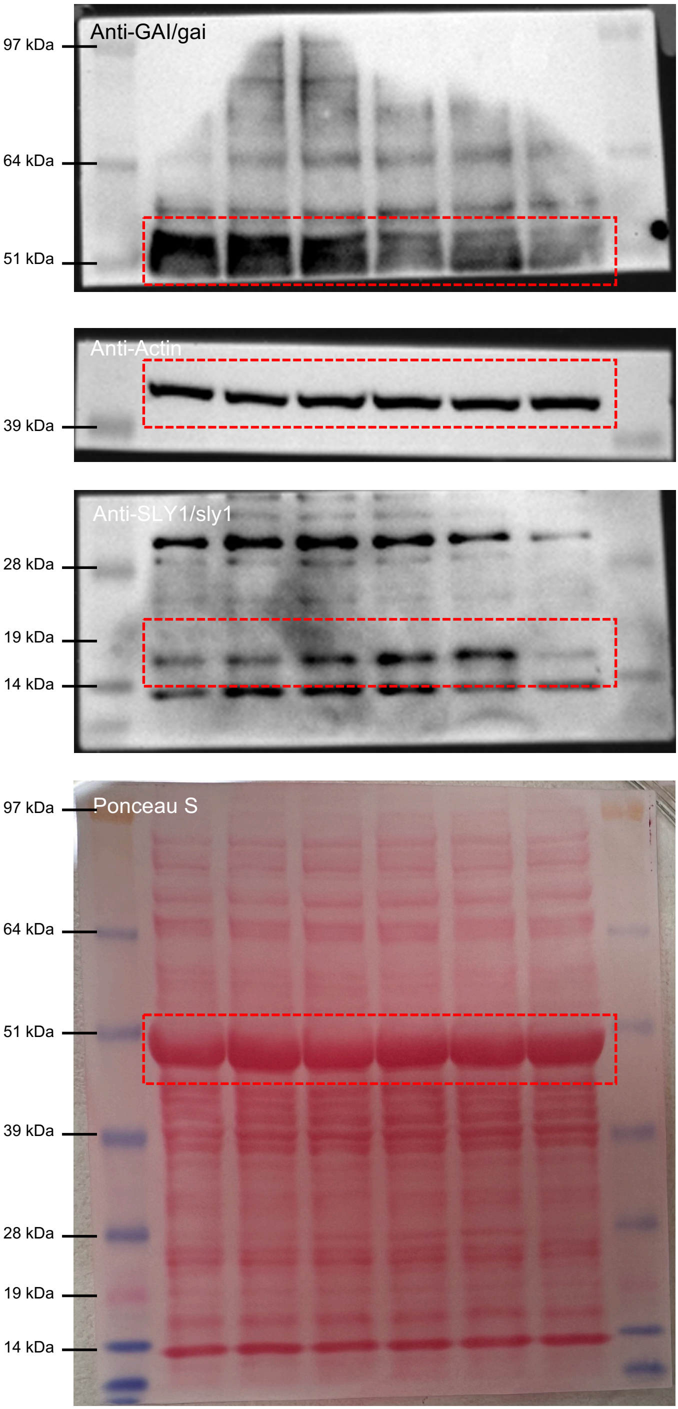

Extended Data Fig. 6c

IP: His anti-MBP

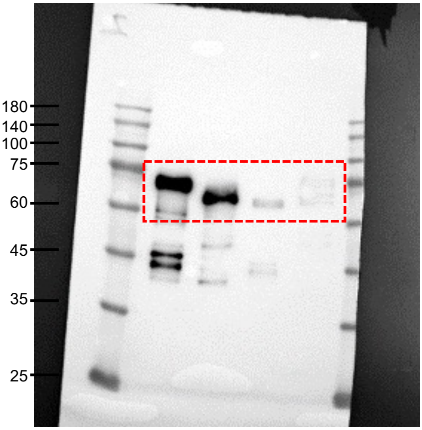

IP: His anti-His

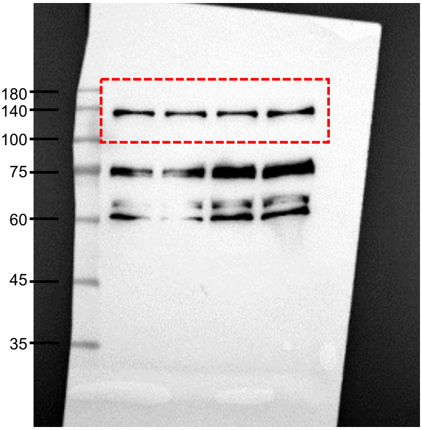

Input anti-MBP

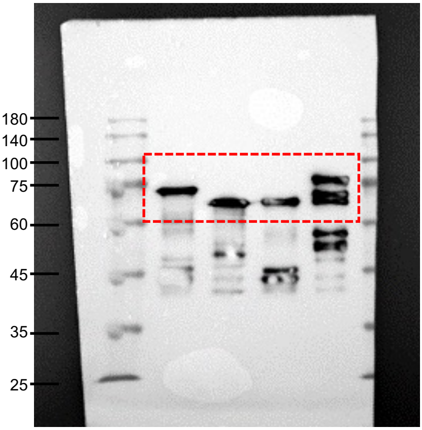

Input anti-His

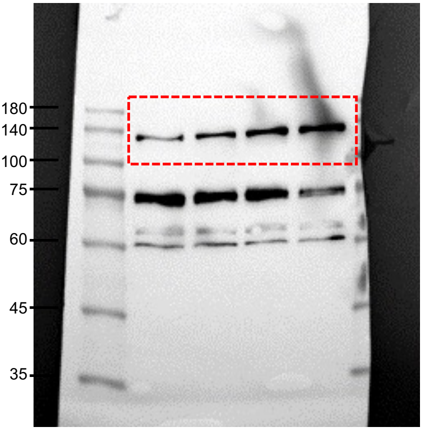

Extended Data Fig. 6d

IP: His anti-MBP

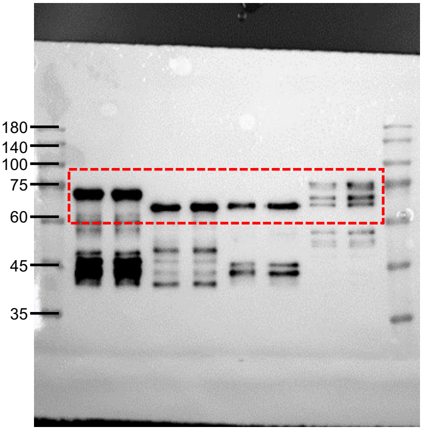

IP: His anti-His

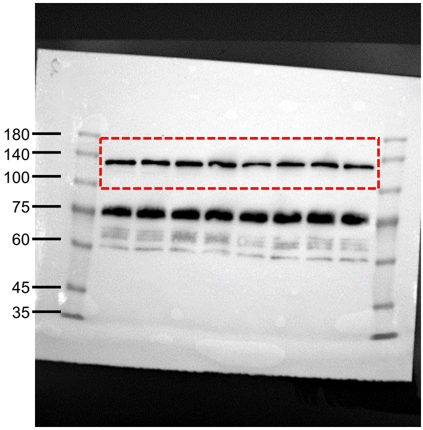

Input anti-MBP

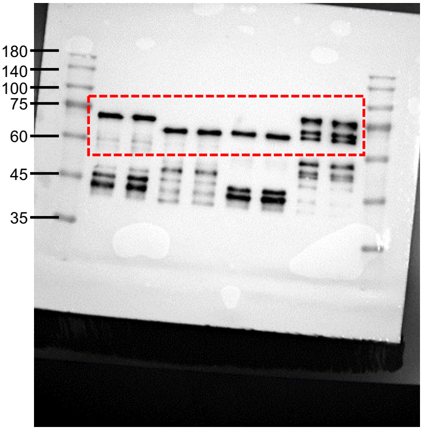

Input anti-GST

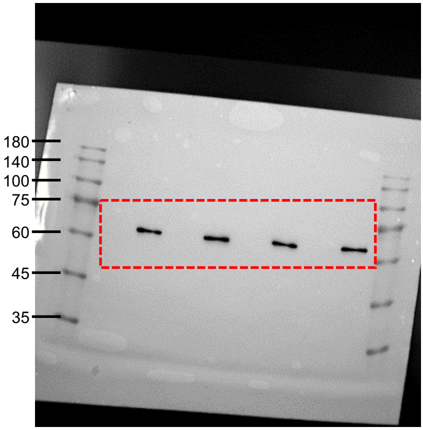

Input anti-His

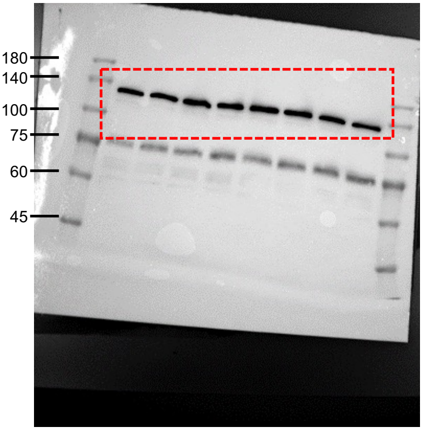

Supplement: Supplementary file 4 — Unprocessed western blots. [file 41477_2023_1556_MOESM4_ESM.pdf]
